# Influence of Fe and Mn on the Microstructure Formation in 5xxx Alloys—Part II: Evolution of Grain Size and Texture

**DOI:** 10.3390/ma14123312

**Published:** 2021-06-15

**Authors:** Jakob Grasserbauer, Irmgard Weißensteiner, Georg Falkinger, Peter J. Uggowitzer, Stefan Pogatscher

**Affiliations:** 1 Christian Doppler Laboratory for Advanced Aluminum Alloys, Chair of Nonferrous Metallurgy, Montanuniversitaet Leoben, Franz-Josef Straße 18, 8700 Leoben, Austria; irmgard.weissensteiner@unileoben.ac.at (I.W.); stefan.pogatscher@unileoben.ac.at (S.P.); 2 AMAG Rolling GmbH, A-5282 Ranshofen, Austria; georg.falkinger@amag.at; 3 Chair of Nonferrous Metallurgy, Department Metallurgy, Montanuniversitaet Leoben, Franz-Josef Straße 18, 8700 Leoben, Austria; peter.uggowitzer@mat.ethz.ch

**Keywords:** aluminum, 5xxx alloys, constituents, dispersoids, microstructure, particle stimulated nucleation, Zener pinning, texture

## Abstract

In recent decades, microstructure and texture engineering has become an indispensable factor in meeting the rising demands in mechanical properties and forming behavior of aluminum alloys. Alloying elements, such as Fe and Mn in AlMg(Mn) alloys, affect the number density, size and morphology of both the primary and secondary phases, thus altering the grain size and orientation of the final annealed sheet by Zener pinning and particle stimulated nucleation (PSN). The present study investigates the grain size and texture of four laboratory processed AlMg(Mn) alloys with various Fe and Mn levels (see Part I). Common models for deriving the Zener-limit grain size are discussed in the light of the experimental data. The results underline the significant grain refinement by dispersoids in high Mn alloys and show a good correlation with the Smith–Zener equation, when weighting the volume fraction of the dispersoids with an exponent of 0.33. Moreover, for high Fe alloys a certain reduction in the average grain size is obtained due to pinning effects and PSN of coarse primary phases. The texture analysis focuses on characteristic texture transformations occurring with pinning effects and PSN. However, the discussion of the texture and typical PSN components is only possible in terms of trends, as all alloys exhibit an almost random distribution of orientations.

## 1. Introduction

With the increasing demands on material recycling, especially in the field of lightweight materials, such as aluminum alloys, a profound knowledge of the effects of increased impurity levels (e.g., Fe) on the property profile of the manufactured products is of essential importance. The properties of the various aluminum alloys should be maintained even with increased content of impurity elements and meet the application requirements [1,2] In the category of AlMg(Mn) (5xxx) alloys, which unify medium strength and good formability, as well as good corrosion resistance, the final properties are largely determined by the solute content of Mg, the cold rolling degree (CRD) and the microstructure [3,4] Additionally, with superior deep-drawing properties, the AlMg(Mn) alloys are widely used in the automotive industry [5,6].

With regard to the influence of secondary alloying elements Fe, Mn and Si, the aluminum alloys show distinct formation of various intermetallic phases [7,8,9] which affect the processing and microstructure of the material [10,11]. For increased iron contents, typical formation of the needle-like Al_3_Fe (or Al_13_Fe_4_) phase is observed [12]. Various studies already accentuated the adverse effects of Fe on the materials properties, which can be partially counteracted by spheroidizing the phases by adding ‘Fe-corrector’ elements, such as Mn [12]. Furthermore, the main secondary alloying element Mn tends to coarsen and spheroidize the constituent Al-Fe(-Si) phases and initiates the formation of secondary phases (dispersoids) of type Al_6_Mn during homogenization [12,13]. These dispersoids play a crucial role in grain size control during subsequent rolling and recrystallization processes [14]. The Si content in the AlMg(Mn) alloys influences both the primary and secondary phase precipitation. During casting, Si can form stable Mg_2_Si precipitates, as well as different types of Al(Fe,Mn)Si phases. Phase transformations and the precipitation of secondary α-Al_15_(Fe,Mn)_3_Si_2_ dispersoids are likely to occur in the following homogenization treatment. Finally, the precipitation kinetics, grain size and, thus, the properties of Mn, Fe and Si containing 5xxx aluminum alloys are also strongly affected by the cooling rate in the casting process. In general, higher cooling rates during casting significantly refine the grain size, as well as constituent phases, and can further change the levels of solute Fe, Mn and Si [15,16,17,18,19]. A comprehensive overview on the details of primary and secondary phases formed in AlMg(Mn) alloys with varying Fe and Mn additions under different processing parameters, such as solidification conditions, homogenization temperature and degree of cold rolling, is given in Part I of this work [15].

With regard to the effects of primary and secondary phases on the recrystallization processes and, thus, on the evolution of microstructure, texture and the final properties, two fundamentally different mechanisms must be taken into account: particle stimulated nucleation (PSN) and grain boundary pinning [14,20,21]. In general, the PSN mechanism occurs in the vicinity of rather large (primary) particles (>1 µm), where the accumulation of dislocations and the formation of recrystallization nuclei are favored. The resulting grain size is then related to the interparticle distances. Moreover, the PSN effect is commonly associated with changes in the typical recrystallization textures of Aluminum alloys [14,20,22,23,24,25]. 

Meanwhile, the influence of small, nanometer sized (secondary phase) particles is described by the Smith–Zener pinning effect [26]. In the case of normal grain growth after primary recrystallization, the grain boundary movement is impeded by pinning forces of small dispersoid particles (“Zener drag”), which depend on the particle sizes, morphologies and volume fractions. Over several decades, numerous research groups improved the original Smith–Zener approach by taking various parameters, such as shape, orientation, or the constitution of the particle-matrix interface, into account [27,28,29,30,31,32,33]. Concerning the impact of dispersoid shape on the pinning efficiency, the authors of [27] derived formulas for the extreme cases of differently oriented spheroidal dispersoids and their restrictive forces on the moving grain boundary. However, the applicability of the formulas to experimentally derived data is still of high complexity.

With the progress in computational modelling in the last forty years, various simulations were performed on the interaction of particles and grain boundaries [34,35,36,37,38,39,40,41,42,43,44,45,46]. The simulation algorithms resulted in a broad spectrum of possible parameters for the formulation of the Smith–Zener pinning. A comparison of experimental data to the various theoretical formulations for the Zener pinning pressure can be performed by the equation for the Zener limiting grain size R_lim_ (1) [28],

(1) $$\frac{R_{\lim}}{r}=\frac{K}{f^{m}}$$

which represents the resulting grain size after recrystallization and grain growth till reaching equilibrium conditions (r, particle size; f, particle volume fraction). Over the years, not only the dimensionless constant K (considering geometric relations of boundary curvature and grain size) was altered in the various approaches, but also the exponent m, accounting for the pinning efficiency of the dispersoids. In general, it was found that for higher volume fractions the exponent m will be <1. However, the exact definition of “higher” volume fractions differs in the various studies and ranges from 1 vol.% up to 3 vol.% of the secondary phase particles [28,47].

In addition to the effects of primary and secondary phases on the grain size, the properties of the final Aluminum sheets are also significantly influenced by the texture of the material [14,48,49,50,51,52]. In typical aluminum rolling processes, the fcc crystal structure and the high stacking fault energy favor the formation of characteristic rolling textures in the deformed state. The orientations Brass {011}<211>, Copper {112}<111> and S {123}<634>, typically referred to as β-fiber [6,53,54,55,56,57], arise from the preferred dislocation slip systems in the material and represent rotations of the crystallites during cold deformation ending in this stable alignment [14].

During subsequent heat treatments, recrystallization, in terms of classical nucleation and growth mechanisms, rearranges the microstructure and the texture of the material. Whereas in earlier years the discussions focused on the differences between two common mechanisms of recrystallization texture formation—oriented nucleation and oriented growth—an implication of both effects is now the preferred approach [52,58]. The main recrystallization texture component in the majority of Aluminum alloys is the Cube orientation {001}<100>, which evolves from remaining Cube nuclei in the deformed state and was the subject of decades of research [49,59,60,61,62,63,64]. Furthermore, frequently found accompanying recrystallization orientations, such as Goss {011}<100> or Q {013}<231>, preferentially nucleate at shear bands, which were also found to occur particularly in AlMg(Mn) alloys [65].

The typical Aluminum recrystallization texture can be altered by the presence of primary and secondary phases, due to the effects of PSN and Zener pinning. In the case of particle stimulated nucleation, the two specific texture components P {011}<122> and Cube_ND_ {001}<310> are generally stated to preferentially evolve, together with a rather weak classical recrystallization texture [24,66,67]. Moreover, the Zener drag exerted by small dispersoid particles impedes the grain boundary movement and therefore changes the recrystallization texture by modifications of the oriented growth mechanisms. The resulting texture is in many cases still dominated by the Cube component, which is least affected by the drag forces due to low energy boundaries and the size advantages of emerging Cube nuclei [25,68]. In case of concurrent appearance of both effects, the Zener pinning dominates the recrystallization texture and effectively suppresses the growth of PSN nuclei [23,66,69].

In terms of the mechanical properties and forming behavior, the texture modifications due to particle related (recrystallization) effects can yield considerable improvements in the final sheet quality. For the AlMg(Mn) alloys, the combination of PSN and Cube texture components can optimize the resulting earing behavior and thus enhance the formability of the 5xxx Al sheets [52]. The number density and distribution of primary and secondary phases can further weaken the Portevin–Le Chatelier (PLC) effect in those alloys [70,71]. Moreover, the PSN components can effectively suppress Cube banding in 6xxx alloys, therefore reducing the probability of the undesired roping phenomenon [6,66,72].

The present study focuses on the microstructure and texture evolution in 5xxx Al alloys with different Fe and Mn levels. A detailed description and discussion of the primary and secondary phase formation in alloys based on Al4.5Mg0.1Si with varying Fe (0.1 and 0.4 wt.%) and Mn (0.2 and 1.0 wt.%) contents is given in Part I of the study [15]. In this Part II, the effects of the mechanisms PSN and Zener pinning are discussed and the experimental data compared to established theories.

## 2. Materials and Methods

**Figure 1:**
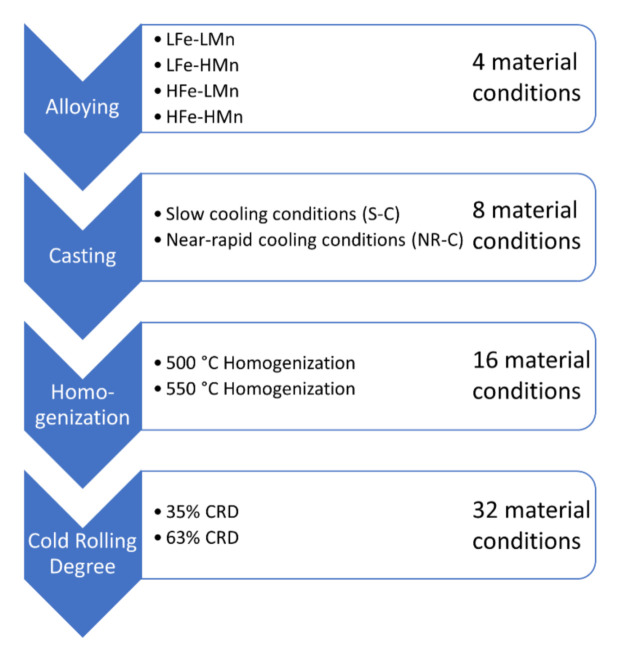
Sample processing scheme, including all variations of the individual process steps.

The studied alloys were based on Al4.5Mg0.1Si with varying Fe (0.1 or 0.4 wt.%) and Mn (0.2 or 1.0 wt.%) contents. Starting from four different alloys (low Fe, low Mn; low Fe, high Mn; high Fe, low Mn; high Fe, high Mn), the variations in casting cooling rate (NR-C: near-rapid cooling, ~50 K/s; S-C: slow-cooling, ~1–2 K/s), homogenization treatment (500 °C and 550 °C) and cold rolling degree (35% and 63%), before final soft annealing (salt bath at 500 °C for 5 min and water quenching), resulted in 32 different sample states (Figure 1). Basically, while the high Fe contents resulted in higher volume fractions of primary phase particles, the high Mn favored the formation of dispersoids and resulted in high volume fractions, especially in the HFe-HMn alloy. Details of the casting and further processing steps of the alloys. as well as the analyses of the primary and secondary phases in terms of their composition, volume fraction and morphology, are presented in Part I of the study [15].

Investigations regarding the microstructure formation, such as grain size and Zener pinning, as well as texture analysis of the final soft annealed state, were performed on cross-sectional samples (looking in the transverse direction (TD) of the cold rolled sheet). Three different experimental methods were used for grain size analysis:

- High contrast backscattered electron (BSE) images from scanning electron microscopy;
- Light optical microscopy (LOM);
- Electron backscattered diffraction (EBSD).

The sample preparation for all characterization methods started with standard metallographic sample preparation, including cutting, embedding, grinding and polishing, plus oxide suspension (OPS, Struers) polishing on a Struers Tegramin 30 grinding and polishing machine. The BSE micrographs were recorded from these samples using a scanning electron microscope (SEM) (JEOL 7200F FEG-SEM, Tokyo, Japan) at an accelerating voltage of 10 kV or 5 kV at higher magnifications for dispersoid analysis. The EBSD measurements were performed on the SEM equipped with the EBSD-measurement system (Nordlys Nano detector, Oxford Instruments, Abingdon, UK) using 20 kV accelerating voltage and a 70° pre-tilt sample holder. Parameters and details on the necessary electropolishing surface treatment for the EBSD analysis can be found in [73]. For light optical microscopy, additional Barker etching of the OPS-polished samples with Barker etchant according to [74] using the Struers Lectro-Pol 5 unit at a temperature of 10 °C and a voltage of 25 V for 50 s accomplished the surface preparation. The LOM micrographs were recorded by utilizing polarized light in a reflected-light microscope Axio Imager M1m (Zeiss, Oberkochen, Germany).

The grain size analysis of the micrographs was carried out performing the line intercept method on LOM and BSE images of various sizes using the free software tool ImageJ. Each measurement contained a minimum of twelve lines (three in rolling direction (RD), three in normal direction (ND); two images per sample), with each line including at least 12 intercept points. Grain size analysis of the EBSD data of was performed by means of the Matlab based toolbox MTEX 5.2.beta3, which is described in detail in [73] and also used the line intercept method on two cross-sectional scans of size 1600 × 1200 µm^2^ per sample (EBSD). The EBSD micrographs are presented in the form of inverse pole figure mappings (IPF) in the RD–ND plane.

The mean grain radius R was calculated from the mean intercept length, taking into account the proportionality constant of 0.75, as described in [75,76]. Grain-defining parameters, such as a minimum grain misorientation angle of 15° and a minimum grain size of 5 µm^2^, as well as grains situated on the sheet or scan edge and coarse primary particles, were considered as exclusion factors.

The calculation of the Zener limiting grain size follows Equation (1), with the parameters K and m relating to the different models. The Manohar model used the parameters K = 0.17 and m = 1 [33], whereas the Smith–Zener model used K = 4/3 and m = 1 [26]. The implementation of the ellipsoidal particle morphology followed the derivations of Ryum [32] and yields the Equations (2) and (3) to calculate the limiting grain radii in RD and ND:

(2) $$\frac{R_{\mathrm{RD}}}{r}=\frac{2}{3f^{m}}{\left(\frac{1}{\mathrm{AR}}\right)}^{0.47}$$

(3) $$\frac{R_{\mathrm{ND}}}{r}=\frac{1}{3f^{m}}\left(1+\frac{1}{\mathrm{AR}}\right){\frac{1}{\mathrm{AR}}}^{0.33}$$

with AR being the aspect ratio of the dispersoids. The calculation again used the exponent m = 1. More information on the mathematical considerations is given in [14,27].

The quantification of texture (using MTEX) focused on the relative intensity of ideal orientations (within 10° maximum deviation) extracted from the EBSD area fractions and normalized by the area fraction of a random orientation distribution, multiplied by the number of the respective symmetric equivalents. The quantification was based on the same cross-sectional scans as the grain size analysis including at least 4000 grains. The ideal orientations of the analyzed components are listed in Table 1.

## 3. Results

This section illustrates the evolution of the characteristic microstructural parameters and the texture as a function of the Fe and Mn content and the processing conditions.

### 3.1. Microstructure Evolution and Resulting Grain Size

As a result of the different conditions during casting, homogenization and cold rolling, a wide distribution of grain sizes of the final soft annealed samples was observed. Figure 2, Figure 3, Figure 4, Figure 5 and Figure 6 depict the microstructures of the LFe-LMn and HFe-HMn alloys by means of different micrographs. The selected sample states show the possible differences of the final microstructure for the casting conditions, the homogenization treatment and the degree of cold rolling. All other sample states and micrographs can be found in the Appendix A (Figure A1, Figure A2, Figure A3, Figure A4, Figure A5, Figure A6, Figure A7, Figure A8, Figure A9, Figure A10, Figure A11, Figure A12, Figure A13, Figure A14, Figure A15, Figure A16, Figure A17, Figure A18, Figure A19, Figure A20, Figure A21, Figure A22, Figure A23, Figure A24, Figure A25, Figure A26 and Figure A27). The general trends between composition, processing and microstructure and texture formation are given in the discussion.

**Figure 2:**
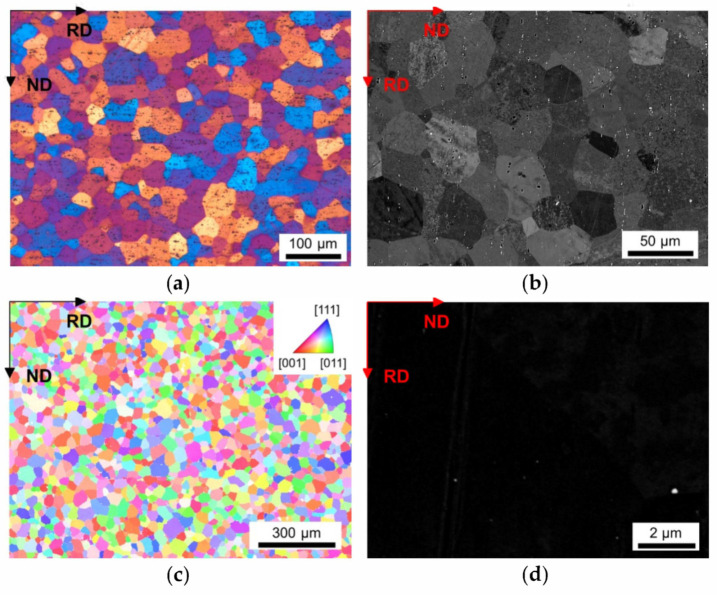
Microstructure of the soft annealed LFe-LMn cast under NR-C condition, homogenized at 500 °C and cold rolled to a CRD of 35%. (**a**) LOM. (**b**) BSE image. (**c**) EBSD IPF map in RD–ND plane. (**d**) BSE micrograph showing dispersoids.

**Figure 3:**
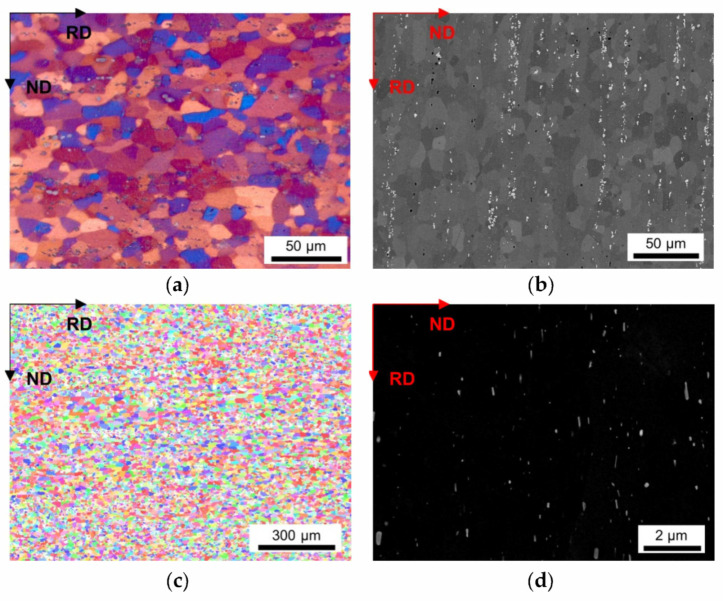
Microstructure of the soft annealed HFe-HMn cast under NR-C condition, homogenized at 500 °C and cold rolled to a CRD of 35%. (**a**) LOM. (**b**) BSE image. (**c**) EBSD IPF map in RD–ND plane. (**d**) BSE micrograph showing dispersoids.

The LOM micrographs of the alloys in Figure 2a and Figure 3a provide a good general overview of the grain morphologies and sizes (note the different map scale bars). While for the LFe-LMn alloy almost equiaxed grains were observed, the HFe-HMn alloy indicates a slight stretch of the grains remaining in the former rolling direction. Furthermore, a significant refinement of the grains can be seen in Figure 3, which is obviously associated with the higher number density of dispersoids. Apart from the dispersoids, the coarse Al-Fe-Mn(-Si) primary phases also seem to affect the microstructure. The BSE images in Figure 2b and Figure 3b show band-wise arrangement of the primary phase precipitates and depict the distinct increase in primary phase fraction with the higher Fe and Mn contents.

**Figure 4:**
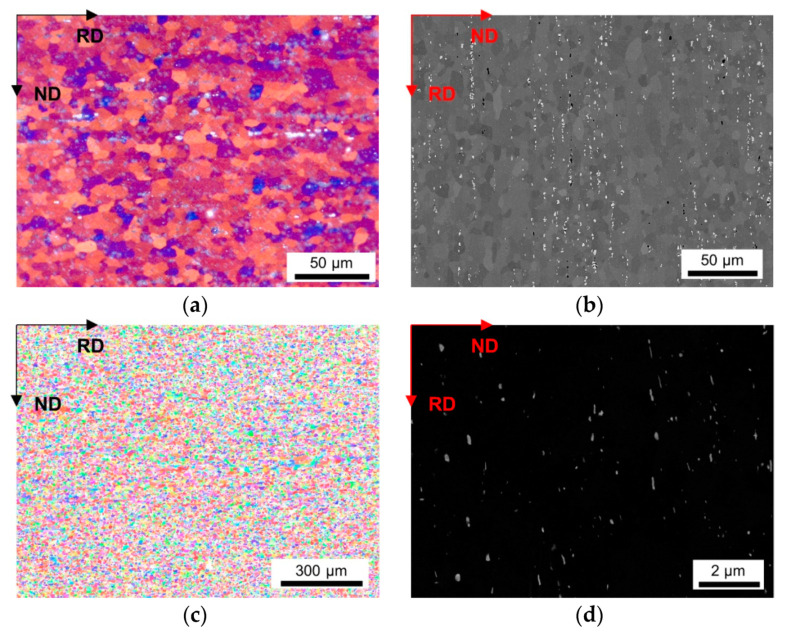
Microstructure of the soft annealed HFe-HMn cast under NR-C condition, homogenized at 500 °C and cold rolled to a CRD of 63%. (**a**) LOM. (**b**) BSE image. (**c**) EBSD IPF map in RD–ND plane. (**d**) BSE micrograph showing dispersoids.

A higher cold rolling degree of 63% causes greater fragmentation and, therefore, refinement of the coarse phases in the HFe-HMn alloy (compare Figure 3b and Figure 4b). The BSE images will further be considered for grain size analysis, since the channeling contrast reveals the size and structure of the grains.

**Figure 5:**
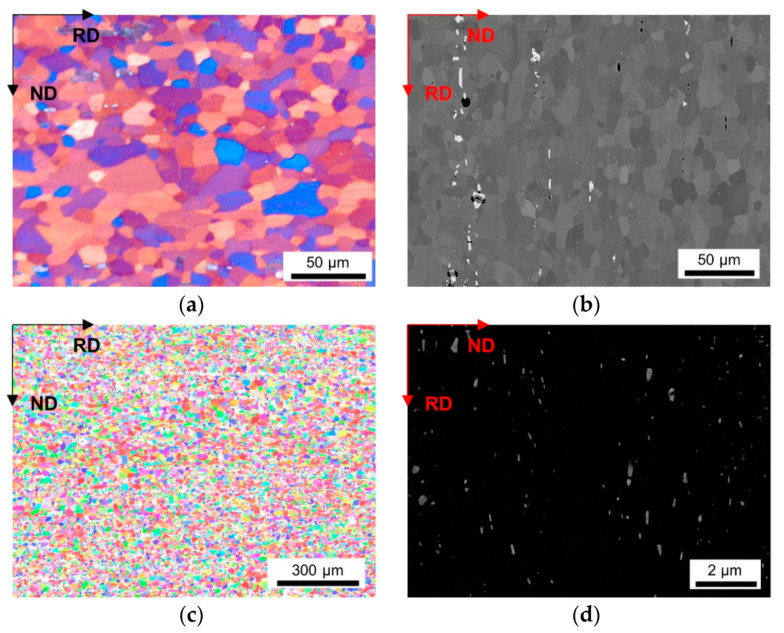
Microstructure of the soft annealed HFe-HMn cast under S-C condition, homogenized at 500 °C and cold rolled to a CRD of 63%. (**a**) LOM. (**b**) BSE image. (**c**) EBSD IPF map in RD–ND plane. (**d**) BSE micrograph showing dispersoids.

A similar refinement as with the CRD is obtained for the NR-C in comparison to the S-C casting conditions for the HFe-HMn alloy (Figure 4a,b and Figure 5a,b). Besides the grains, the primary phases in particular are distinctly coarsened in the S-C casting samples (Figure 5b).

**Figure 6:**
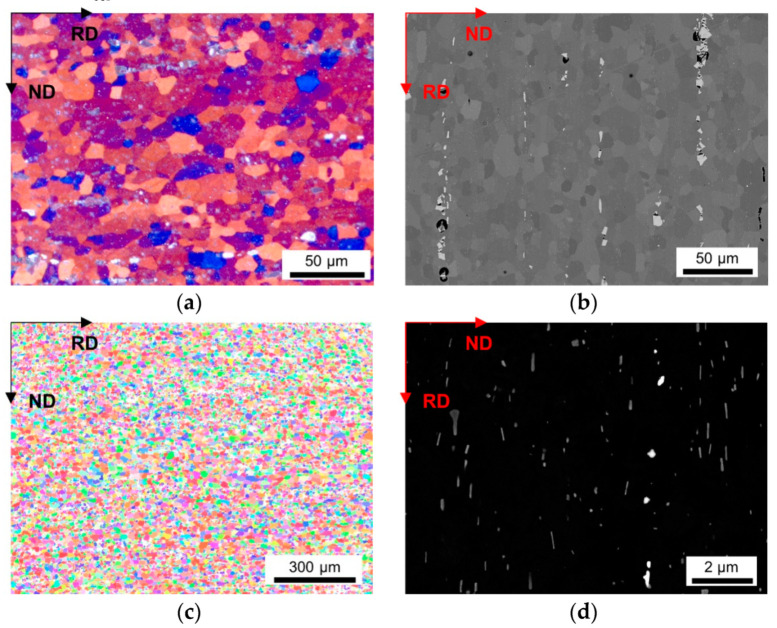
Microstructure of the soft annealed HFe-HMn cast under S-C condition, homogenized at 550 °C and cold rolled to a CRD of 63%. (**a**) LOM. (**b**) BSE image. (**c**) EBSD IPF map in RD–ND plane. (**d**) BSE micrograph showing dispersoids.

The inverse pole figure (IPF) maps of the presented figures indicate the absence of preferred orientations in both LFe-LMn and HFe-HMn samples (Figure 2c and Figure 3c). Furthermore, none of the processing conditions show significant alterations of the resulting grain orientations as the random texture character is preserved as shown for the HFe-HMn alloy in Figure 3c, Figure 4c, Figure 5c and Figure 6c. The color code legend inserted in Figure 2c applies to all IPF maps given in this study.

The typical dispersoid distribution and arrangement of the alloys is shown in Figure 2d, Figure 3d, Figure 4d, Figure 5d and Figure 6d. While the low Mn containing alloy is nearly free of secondary phase particles (Figure 2d), the HFe-HMn shows spherical, as well as rod- or plate-shaped, dispersoids (Figure 3d). Similar to the findings in Part I [15], the nanometer sized particles align with the rolling direction. As explained later, the preferred orientation of the dispersoids results in unequal Zener pinning forces for RD and ND growth directions, which is implemented in some of the calculations of the Zener limiting grain size.

As described in detail in Part I, neither the processing conditions as the given CRD (compare Figure 3d and Figure 4d), nor the casting cooling conditions (compare Figure 4d and Figure 5d) cause distinct alterations in the final dispersoid volume fraction and morphology. However, coarsening and more rod- or plate-shape morphology of the dispersoids is obtained for the higher homogenization temperature of 550 °C (compare Figure 5d and Figure 6d).

The resulting grain sizes for the various 63% CRD sample states are listed in Table 2, including the data for the different characterization methods—LOM, BSE and EBSD. Because of the unbalanced aspect ratios for most of the samples, the results are given in terms of grain radii derived from line intercept measurements in RD, as well as ND, for all micrographs.

The data given in Table 2 clearly show the influences of the different characterization methods. While the numbers of the mean grain radii from the LOM and BSE micrographs, R_LOM_ and R_BSE_, are about the same, the mean grain size derived from EBSD measurements, R_EBSD,_ is significantly lower. Since EBSD measurement and processing are the least user affected, the true value will be closer to the numbers obtained for R_EBSD_. The higher measurement errors for EBSD grain sizes result from different considerations than for LOM and BSE. For LOM and BSE images the measurement error (standard deviation) is given by the standard deviation of the mean value resulting from the measurements of the three different images, while the standard deviation for the EBSD data comprises the data of all individual grains. The basic discussion of the subsequent microstructure evolution will therefore be based on grain parameters obtained with the EBSD measurements.

Table 2 clearly illustrates the influence of sample processing on grain morphologies, i.e., the effects of casting cooling rate, homogenization and cold rolling. For example, the NR-C cast samples show significantly finer grain structures in the final annealed sheets. Moreover, the faster cooling also results in a more balanced aspect ratio for most of the grains. The homogenization heat treatment results in a slight alteration of the microstructure since samples show reduction or increase in the mean grain radius with the higher maximum homogenization temperature of 550 °C in comparison to 500 °C. 

Information on the resulting grain sizes for sample processing with a CRD of 35% is given in Table 3, where the comparison to Table 2 reveals the influence of the cold rolling degree.

The alloying elements Fe and Mn differently impact the final microstructure. In general, the largest grains were observed for the LFe-LMn alloy. In the absence of dispersoid particles and with the low number density of primary phases, the recrystallization and grain growth are not effectively suppressed, resulting in grain sizes of around 20–25 µm for the S-C casting conditions.

With the increased Mn level in the LFe-HMn alloy and the strong formation of dispersoid particles, the grain growth is significantly retarded. Comparing the grain sizes in Table 2, the average grain size decreases by more than a factor of 2, depending on the sample processing; however, as observed for the HFe-HMn alloy, the change in Fe contents with the high Mn level does not significantly alter the grain radii. Clearly, in the presence of high dispersoid density, the higher number density of the primary phases does not show any considerable effects.

Contrarily, the HFe-LMn alloy exhibits a refinement of the grains compared to the LFe-LMn alloy. Since the number density of dispersoids is comparably low in both alloys, the effect may be related to the higher number density of primary phase particles in this sample.

### 3.2. Zener Pinning Effect of Dispersoids

The importance of the Zener pinning effect is highlighted with the large differences in the resulting grain sizes shown in Table 2. With the dispersoids formation and the experimentally observed variations in the number density after homogenization (see Part I [15]), a wide range of the resulting pinning forces is to be expected. For accurate calculations of the limiting grain size, the volume fractions, as well as mean radii and aspect ratios of the dispersoids, were analyzed in all 32 different final sample states. Table 4 and Table 5 show the results of the analyses for the different alloys with NR-C cast conditions and 63% CRD at 500 °C and 550 °C homogenization, respectively.

Comparing the volume fractions of the secondary phase particles of the four alloys in Table 4, the trend of favored dispersoid formation in the high Mn alloys is obvious, whereas the Fe contents seem to have only little influence. Furthermore, the aspect ratio of the dispersoids is clearly more unbalanced in the higher volume fraction containing alloys LFe-HMn and HFe-HMn. Similar trends were observed for the samples with higher homogenization temperature (Table 5), although the volume fraction of dispersoids is significantly lower in the LFe-HMn alloy compared to HFe-HMn. Distinct coarsening of the secondary phase particles was observed with the higher homogenization temperature comparing the values of the mean radii in Table 4 and Table 5, whereas the aspect ratios remained almost unaffected, especially for the LFe-HMn and HFe-HMn alloys.

Table 4 and Table 5 also contain the results of the Zener limiting grain size calculated according to Equation (1) with the experimentally evaluated particle sizes and volume fractions and different parameters from literature (see Section 2). Comparing the grain size predictions of the different models in Table 4, a wide range of possible values is obtained. Concerning the alloys containing low dispersoid volume fractions LFe-LMn and HFe-LMn, the approaches of Smith–Zener and Ryum both distinctly overestimate the resulting mean grain size in comparison to the experimentally obtained data in Table 2.

The calculations using the parameters of Manohar tend to accord with the mean grain radii from EBSD measurements, especially for the HFe-LMn alloy. Similar results are obtained for the higher homogenization temperature of 550 °C in Table 5, where the approach of Manohar roughly fits for LFe-LMn and HFe-LMn. 

On the other hand, the calculations for LFe-HMn and HFe-HMn show contrary results. For the 500 °C homogenization temperature, the models of Smith–Zener and Ryum yield results which are in good accordance with the experimentally observed data.

However, the grain size calculations using the ellipsoidal model of Ryum show better conformity for ND than for RD. Again, very similar relations were observed for the 550 °C homogenization in Table 5, although the calculated grain sizes are slightly higher than the average experimentally obtained data. The larger resulting grain size for the higher homogenization temperature can be attributed to the higher average radius of the dispersoids. Furthermore, the poor comparability of the resulting grain radii from Manohar’s approach is noticeable for the two high manganese containing alloys.

Further experimental data can be found in Table A1 and Table A2 in the Appendix A, comparing other processing conditions, such as S-C cast and cold rolling degree of 35%.

**Figure 7:**
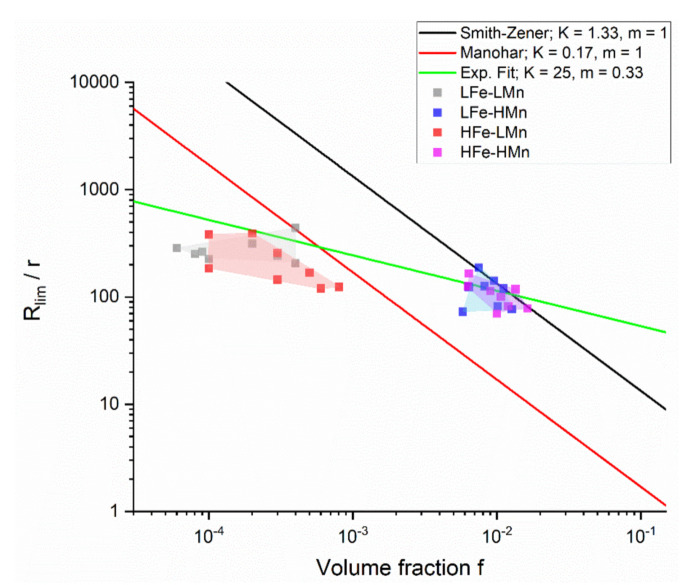
Comparison of the Zener limiting grain size calculated from the models of Smith–Zener and Manohar [26,28,33] to the experimentally observed data for the four different alloys, including the best fit.

The different Zener models and the effects of various parameters on the limiting grain radius in dependence on the dispersoid radius and the total volume fraction of the secondary phase particles are illustrated in Figure 7. The experimental data points include the volume fractions and radii of the dispersoids, as well as the grain radii (from EBSD), of all sample states of the four alloys.

For the samples with low dispersoid volume fraction LFe-LMn and HFe-LMn, all models overestimate the experimentally obtained resulting grain size, but the analytical description according to Manohar is clearly better than that according to Smith–Zener. Conversely, the Smith–Zener model describes the measured mean grain sizes of the samples with high dispersoid volume fraction LFe-HMn and HFe-HMn quite well.

Numerous computational 3D simulations on the Zener limiting grain size derived values of the exponent m close to 0.33 for dispersoid volume fractions over 1% [34,35,36]. Figure 7 includes an optimized model fitting the experimental data. Although in fact only applicable for samples with high dispersoid volume fractions LFe-HMn and HFe-HMn, the constant factor K was adapted to best fit the data points for all alloys using m = 0.33. In general, a good conformity can be obtained for LFe-LMn, LFe-HMn and HFe-HMn; only in HFe-LMn is the obtained grain size in average lower than that predicted by the applied model.

### 3.3. Grain Boundary Pinning by Primary Phase Particles

Besides the grain refinement observed in the high dispersoid fraction containing alloys LFe-HMn and HFe-HMn, the HFe-LMn alloy shows significant reduction in the mean grain radius in comparison to LFe-LMn. Since the number fraction of secondary phase particles is comparably low in both alloys, the effect must be connected to the higher Fe content, equivalent to higher primary phase fractions.

**Figure 8:**
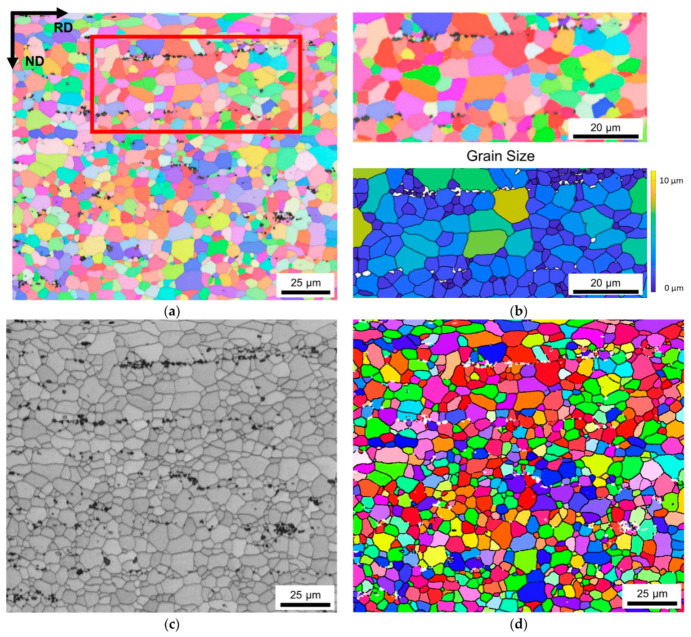
(**a**) Inverse pole figure map in RD–ND plane of NR-C cast HFe-HMn alloy, homogenized at 500 °C and cold rolled to a CRD of 63%. (**b**) Magnified area showing the primary phase arrangement and the area equivalent grain radius of surrounding grains, respectively. (**c**) Band contrast image indicating the fully recrystallized microstructure. (**d**) Overlay of high angle grain boundaries (>15°, black) on the IPF map shown in (**a**).

Figure 8 shows the EBSD inverse pole figure map of NR-C cast HFe-HMn alloy, homogenized at 500 °C and cold rolled to 63%. While Figure 8a provides a more general overview of the microstructure and orientation distribution in the present alloy, Figure 8b gives more details on the primary phases in the marked area (red rectangle). The magnified IPF map (Figure 8b top) shows the dark appearing primary phases in band-wise arrangement situated at the edges of various grains. Closer consideration reveals many of these rather coarse particles located at triple points, already suggesting an impact on the grain boundary mobility and, therefore, the grain growth behavior. Although the suggested Zener pinning of coarse particles is low in comparison to the dispersoid pinning, the general pinning efficiency is increased with the particles situated at grain junctions. Therefore, for the high primary phase volume fraction containing alloys HFe-LMn and HFe- HMn, an additional pinning pressure is obtained resulting in grain refinement, especially for HFe-LMn. The grain size map in Figure 8b (bottom) depicts the color-coded grain size in the magnified area. From both the IPF and the size area map, a certain refinement of the average grain size is obtained around primary phases.

Figure 8c,d show the band contrast and high angle grain boundaries (>15° misorientation) of the investigated sample section. Both indicated fully recrystallized microstructures and do not show remaining subgrains.

### 3.4. Texture Modifications by Primary and Secondary Phase Particles

The texture analysis concerns the orientation data from the EBSD measurements of the total cross sections of the samples including, in all cases, more than 4000 grains. With very low maximum intensities in the calculated orientation distribution functions, the results are presented in terms of bar charts on the EBSD area fractions of selected ideal texture components. Additionally, Figure A28 exemplary shows the ODF data of a HFe-HMn alloy plotted in sections of Euler angle φ_2_, including the ideal orientations of specific texture components. In general, as already observable in the IPF maps in Figure 2, Figure 3, Figure 4, Figure 5 and Figure 6 and Figure 8, the grains do not show preferred orientations. No peculiarities regarding the interaction of differently sized particles with the surrounding grain orientations can be seen.

**Figure 9:**
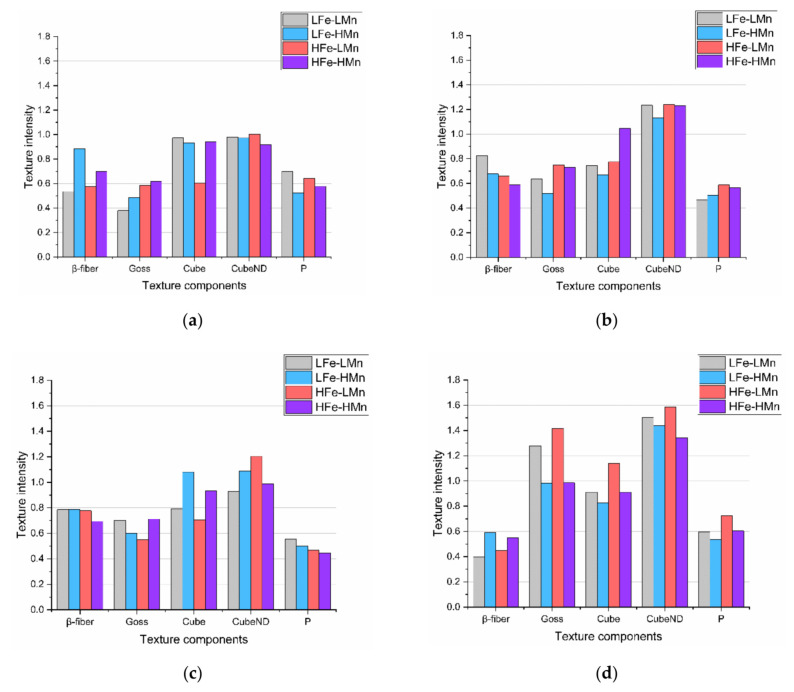
Influence of the processing parameters on the resulting texture of the different alloys in the final soft annealed sheets. (**a**) S-C cast, 500 °C homogenized and 35% CRD. (**b**) NR-C cast, 500 °C homogenized and 35% CRD. (**c**) NR-C cast, 550 °C homogenized and 35% CRD. (**d**) NR-C cast, 550 °C homogenized and 63% CRD.

The influence of the various process parameters on the overall texture evolution in the different alloys is shown in Figure 9 (and Figure A29 for other sample states). The resulting texture for NR-C and S-C cast conditions (500 °C homogenization, 35% CRD; Figure 9a,b) demonstrates a generally low impact of the different casting conditions on the final texture. Besides the very low intensities obtained for both the rolling and recrystallization components, the NR-C samples show higher Cube_ND_ texture intensities for the different alloys on average (comparing Figure 9a,b).

The results obtained for the different homogenization temperatures (comparing Figure 9b,c) show a slightly higher persistence of the β-fiber rolling components, as well as a slightly higher content of the classical Cube and Cube_ND_ recrystallization textures for higher annealing temperatures.

In contrast to the casting and homogenization process, the degree of cold work prior to final sheet annealing controls the texture evolution of the sample in some part (comparing Figure 9c,d). Concerning the β-fiber components, Figure 9d clearly shows the weakened intensities obtained for higher cold rolling. As expected, contrary behavior is obtained for the recrystallization components and the Goss orientation.

Furthermore, the texture evolution for the alloys and, thus, the different primary and secondary phase fractions can be derived from Figure 9 and Figure A29 for the various processing parameters. An overall trend of retained β-fiber components in the final sheets was observed with the different secondary phase volume fractions of the alloys. With increasing dispersoid number density the rolling components favorably persist the annealing treatment. 

The Goss component, which can be found in typical rolling and recrystallization textures, exhibits an ambiguous progress, as the intensity is weakened or reinforced in the LFe-HMn and HFe-HMn alloys. The frequently observed Cube component shows low area fractions, which are on average slightly increased for the alloys with higher dispersoid volume fractions. The formation or growth of PSN component P is promoted in some of the LFe-LMn and HFe-LMn alloys. No effects of PSN can be seen in the very erratic CubeND area fractions. For a more detailed analysis of possible PSN effects, an additional evaluation of the areas around coarse primary phase particles was carried out and the occurring texture components analyzed. While the grain size was significantly reduced in the immediate vicinity of the particles (Figure 8b), no clear trend towards intensification of characteristic PSN texture components was obtained.

## 4. Discussion

We first focus the discussion on the resulting mean grain size in the different sample states, which is depicted in Figure 2, Figure 3, Figure 4, Figure 5 and Figure 6 and stated in Table 2, for 63% cold rolled samples, or Table 3, for 35% cold rolled samples, respectively.

Comparing the different results for the line intercept method from LOM, BSE and EBSD micrographs in Figure 2a,c, the importance of well-designed grain size analysis becomes clear. Although LOM and BSE imaging and evaluation are more easily executed, the EBSD technique is less user-biased and was reported in [78] to have advantages especially in the detection of smaller sized grains. This is verified by the results in Table 2, where the average grain size is significantly smaller for experimentally observed data by the EBSD technique. Therefore, especially for further calculations, the usage of EBSD is beneficial to avoid error propagations [78]. However, the standard deviation is higher for EBSD results.

The influence of the processing parameters on the final grain size is denotable from Table 2 and the respective figures. The refinement of the grains and the microstructural features obtained for the NR-C (near-rapid cooling) casting conditions [17] (comparing Figure 4 and Figure 5) will improve the mechanical properties of the Al sheets, since the smaller average grain size is still preserved in the final sheet, to some extent [14]. Furthermore, the combination of the solidification rate and Fe, Mn and Si alloying contents determine the size, shape and composition of the primary phase particles [7,8,9,12] With the high number density of rather small and blocky Al-Fe-Mn(-Si) precipitates, as in, for example, the HFe-HMn alloys (Figure 3 and Figure 4b), the NR-C conditions can reduce the adverse effects caused by stress concentrations of needle-shaped Fe bearing phases and thus increase the mechanical properties, such as r value, yield strength or ultimate tensile strength [11,67].

The comparison of Table 2 to Table 3 clarifies the impacts of the cold rolling degree on the resulting grain size. According to the classical recrystallization theory, the higher pressure for recrystallization in the 63% cold rolled alloys leads to distinct refinement of the microstructure in the soft annealed state (Figure 3 and Figure 4) and might therefore improve the mechanical properties [14].

**Figure A6:**
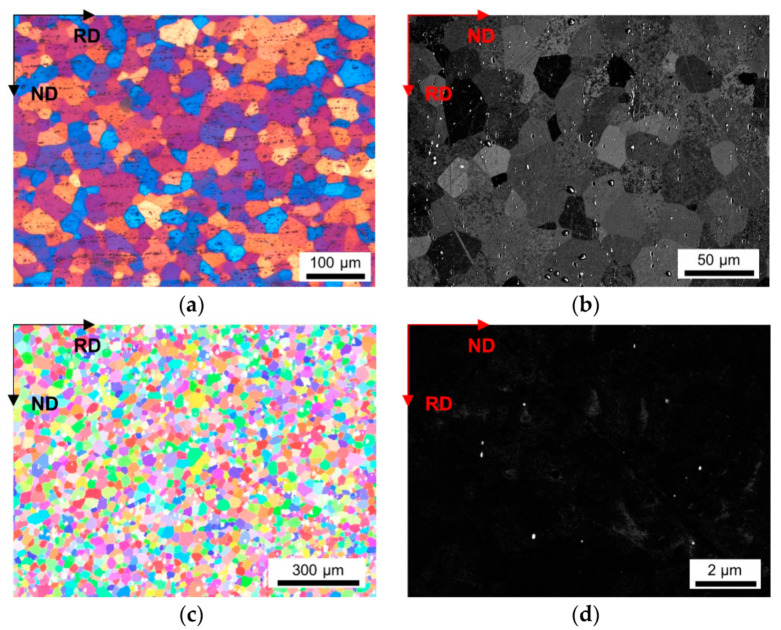
Microstructure of the soft annealed LFe-LMn cast under NR-C condition, homogenized at 550 °C and cold rolled to a CRD of 35%. (**a**) LOM. (**b**) BSE image. (**c**) EBSD IPF map in RD–ND plane. (**d**) BSE micrograph showing dispersoids.

**Figure A28:**
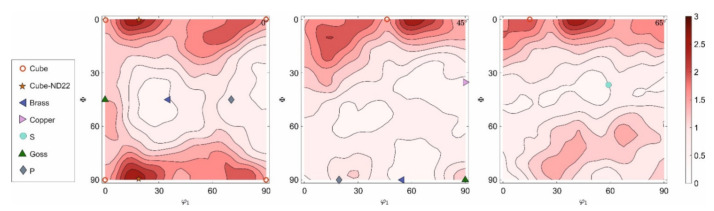
ODF data of the soft annealed HFe-HMn cast under NR-C conditions, homogenized at 500 °C and cold rolled to a CRD of 63% plotted in sections of φ_2_ and including the ideal orientations of specific texture components; left: φ_2_ = 0°, middle: φ_2_ = 45° and right: φ_2_ = 65°.

The effect of the homogenization temperature on the final sheets microstructure is discussed in light of Table 2, as well as Table 4 and Table 5. As reported in Part I of the present study, the shape and constitution of the primary Al-Fe-Mn(-Si) and Mg_2_Si particles alter with the high temperature heat treatment [15,79,80]. Furthermore, secondary phase particles undergo distinct coarsening at the higher homogenization temperature for LFe-HMn and HFe-HMn alloys (see Figure 2 and Figure A6d, as well as Figure 5 and Figure 6d; compare Table 4 and Table 5) [13,79]. Although the results in the average grain size for 500 °C and 550 °C homogenization in Table 2 are ambiguous concerning the exerted pinning forces by the dispersoids, the homogenization treatment plays a fundamental role in the microstructure evolution in multiphase materials, as it predominantly initiates the dispersoid formation [16,79,80].

Alongside the processing parameters, the Fe and Mn alloying levels are crucial for the microstructure evolution in deformation and recrystallization processes [14,67]. Concerning the results in Table 2 or the micrographs given in Figure 2 and Figure 3, either high Fe and/or Mn contents yield significant grain refinement for the HFe-HMn in comparison to the LFe-LMn alloy. However, in reference to the primary and secondary phase volume fractions obtained for the different alloys [15], the effect of Zener pinning requires consideration and sophisticated discussion in this context [10,14,66].

With the formation of Al_6_(Fe,Mn) dispersoids during homogenization found mainly in the high Mn containing alloys, the effective Zener pinning is clearly denoted by the obvious reduction in the average grain size. Besides the verification of the general Zener pinning effect [26], the results for the limiting grain size given in Table 4 and Table 5 vary significantly with the different model parameters for Equation (1) [26,32,33]. The large discrepancies between the approaches of Smith–Zener or Ryum and Manohar occur from the differently chosen pinning and driving pressures for grain growth [28]. However, while [28] stated the good applicability of the Manohar model parameters [33] to predict the resulting grain size in materials with dispersoid volume fractions below f_V_ = 0.05, the data for LFe-HMn and HFe-HMn in Table 4 and Table 5, as well as Figure 7, show best conformity with the original Smith–Zener approach [26].

Although the grain size predictions by the Ryum model [32] do not match the experimentally observed data as well as the Smith–Zener approach, the implementation of the dispersoids ellipsoidal shape in Equation (1) seems a necessary improvement for the present study. With the often obtained rod- or plate-like shape of the dispersoids in Mn and Fe containing 5xxx alloys [13,19,79].and their (banded) alignment with RD in subsequent processing (Figure 2 and Figure 3d), the resulting Zener drag will be different for the individual sample directions [27,35,41,45] The aspect ratios of the dispersoids in the high Mn containing alloys given in Table 4 and Table 5 affect the pinning efficiency in RD and ND and further cause the discrepancies in the grain size in those directions [14].

Concerning the resulting grain size in the LFe-LMn and HFe-LMn alloys, Figure 7 clearly depicts that large discrepancies occur between the predictions of the Smith–Zener or Manohar model and the experimental data. On the other hand, the tentatively applied optimized fit, which follows model parameters obtained for computational studies using the exponent m = 0.33, shows high accordance to the experimental data. Nevertheless, the close match with LFe-LMn and HFe-LMn is unexpected, as the computational model is stated to be valid only for high volume fractions of dispersoids f_V_ > 0.01 [34,35,36]. Moreover, the reliability of the fit is questionable with the high value of K = 25, since most studies proposed this parameter to be around 4/3, as in the original Smith–Zener approach, or even lower [28]. Possible explanations for the deviations in the grain size prediction for the low Mn containing alloys might be that the final soft annealed samples (even though fully recrystallized) either (i) did not reach the static limiting grain size by grain growth in the short-term soft annealing, or (ii) additional (primary phase) particle-related effects reduced the resulting grain size [14].

As shown in Figure 8b for the HFe-HMn alloy, the final microstructure and grain size shows distinct influence of micron-sized fragmented primary phases. With the effect of particle stimulated nucleation, preferential nucleation around these fragmented phases may be partly responsible for grain refinement [14,20,22,25]. However, since the coarse primary particles are favorably situated on grain boundaries and junctions of the final recrystallized grains, they should also be considered for pinning effects during recrystallization and grain growth. Because the particle pinning efficiency decreases with increasing particle dimensions, additional pinning effects are only obtained for high primary phase volume fraction containing alloys HFe-LMn and HFe-HMn [26,27,28]. Therefore, the refined grains for HFe-LMn in comparison to LFe-LMn in Table 2 may be the result of combined effects of PSN and primary phase pinning. The similar trends of grain size reduction for S-C cast HFe-HMn in comparison to LFe-HMn suggest equivalent mechanisms; however, with the high fraction of pinning dispersoids weakening the effect of the primary phases, the difference in the resulting grain size is significantly smaller [69].

In general, the present study verifies the theories of Zener pinning by second phase dispersoid particles. Despite numerous sophisticated corrections and improvements that have included various parameters into the model for the resulting limiting grain size, the applicability for predicting the resulting grain size in experimental or industrial processes is still limited. Even though the present results confirm the conclusions of the original Smith–Zener relation, further investigations and combined implementation of various particle-grain boundary related effects in an all-encompassing model equation are still sought [14,26,28,32].

**Figure A29:**
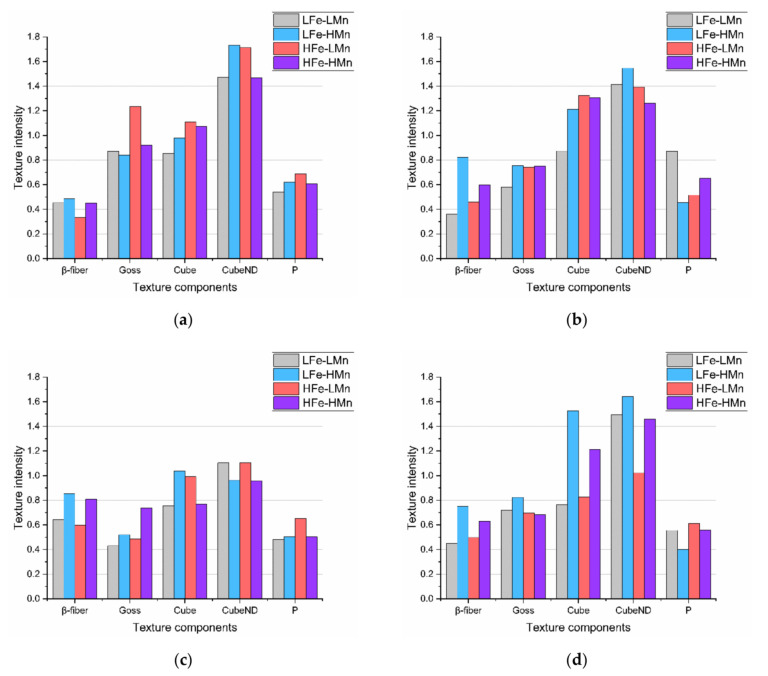
Rolling and recrystallization texture components for the samples states not included in Figure 9. (**a**) NR-C cast, 500 °C homogenized and 63% CRD. (**b**) S-C cast, 500 °C homogenized and 63% CRD. (**c**) S-C cast, 550 °C homogenized and 35% CRD. (**d**) S-C cast, 550 °C homogenized and 63% CRD.

We now discuss the texture evolution of the investigated alloys shown in the IPF maps in Figure 2, Figure 3, Figure 4, Figure 5 and Figure 6c, as well as Figure 9 and Figure A29. All samples exhibit remarkably low fractions of typical aluminum recrystallization textures in the annealed condition. These overall minor intensities were similarly found for laboratory and industrially processed EN AW-5182 alloys [73]. The texture transformations to higher random fractions can be attributed to shear band formation, which is likely in high Mg containing Al alloys implicating Cube texture suppression and preferential nucleation of Goss, P and Q orientation, or to PSN effects [14,58].

Figure 9a,b highlight the small and ambiguous influence of the casting process on the final texture. The variation in the casting cooling conditions S-C and NR-C mainly influences the refinement of the primary phase and the casting grain and accompanying texture modifications will be observed in the comparison of the individual alloys. As both casting processes do not involve any special operations or directed solidification, the formation of pronounced texture components in the as-cast state is not promoted [21] and, thus, the transfer of orientations, such as Cube, to the final soft annealed states is unlikely to occur [54].

The influence of the maximum homogenization temperature on the resulting texture is hardly perceptible when comparing Figure 9b,c. Although grain boundary pinning by dispersoid particles in general affects the texture evolution, the differences in average dispersoid size and amount at 500 °C and 550 °C homogenization do not produce significant texture transformations [23,25,68,69].

It is well established that cold rolling significantly influences the recrystallization behavior of materials by altering the driving pressure for primary recrystallization and normal grain growth [14]. The results on the impact of the CRD on the recrystallization texture (comparing Figure 9c,d) are in line with expectations, as the higher CRD of 63% promotes recrystallization during soft annealing and, hence, exhibit lower fractions of remaining β-fiber components but more pronounced recrystallization textures [14].

With the generally weak texture found in the present study, the texture modifications due to primary particles and dispersoids can only be assessed by trends and not by significant differences in the intensities. The impacts of the Zener drag on the recrystallization texture can be deduced from the behavior of the different alloys in Figure 9 and Figure A29. The weaker transformation of the rolling components in the LFe-HMn and HFe-HMn alloys can be related to pinning effects by dispersoids, since the growth of emerging recrystallization nuclei with Goss, P and Cube_ND_ orientation is retarded [25,66,68,69]. In turn, the intensities of those components are rather low in comparison to the LFe-LMn and HFe-LMn alloys. Furthermore, as the growth of Cube oriented grains is assumed to be least affected by pinning effects, slightly higher Cube fractions are obtained for some of the LFe-HMn and HFe-HMn alloys in various sample states, in agreement with the mechanisms described in literature [25,68,69].

For the formation of the recrystallization structures, the PSN mechanism also needs to be taken into account, as the occurring fragmentation of primary phase particles during rolling can significantly increase the number of potential PSN nuclei (Figure 2, Figure 3, Figure 4, Figure 5 and Figure 6b), thus altering the resulting texture [21]. The PSN related texture component P was slightly more favorably observed for the low Mn alloys; in the case of the high Mn alloys, however, the expansion of the P-oriented nuclei is likely to be retarded by dispersoid pinning effects [23,24,25,68,69]. In general, it has to be emphasized that due to the overall weak textures the change of the annealing texture towards a higher random fraction of orientations through PSN effects is very weakly pronounced [6,58,59].

With the large varieties in primary and secondary phase fractions in the four different alloys and the differences in the resulting grain size, the mechanical properties, as well as the forming behavior, will be affected. In addition to the commonly known increased strength with the reduction in the average grain size, recent studies also state that there can be beneficial effects of (coarse) Fe and Mn bearing primary phases on the mechanical properties, as well as the deep drawability, as the phases can beneficially refine the soft annealed grain structure and favor texture randomization [3,11,67] Moreover, the detrimental effect of Lüdering in the AlMg(Mn) alloys can effectively be reduced by the control of the primary and secondary phase number density, size and total volume fraction [70,71].

Furthermore, whereas numerous publications mention the essential effects of PSN to reduce the mechanical anisotropy or surface effects, such as roping in other Al alloys series [6,23,24,66] the high fraction of random texture components obtained in the 5xxx series will minimize the plastic anisotropy in the material [52,73]. In light of these findings, in particular the Fe and Mn rich alloy HFe-HMn processed via NR-C is expected to exhibit interesting mechanical properties due to the particular particle distribution, small grain size and random annealing textures.

## 5. Conclusions

The present work investigates the influence of Fe and Mn bearing primary and secondary phase particles under various processing conditions on the resulting microstructure and texture. With regard to the findings in Part I on the details on primary and secondary particle characteristics, the following conclusions can be drawn.

- Effective Zener pinning was observed for Al_6_(Fe,Mn) containing high Mn alloys resulting in distinct refinement of the grains in soft annealed samples.
- Rapid cooling conditions in casting and higher cold rolling degrees significantly refine the final soft annealed microstructures in terms of grain size and fragmentation of primary phases.
- The impact of the maximum homogenization temperature on the resulting microstructure is rather small; despite the fact that homogenization is essential for the formation of the secondary phase, i.e., the dispersoids.
- All investigated conditions showed very weak textures in 5xxx alloys. Nevertheless, trends of the influence of CRD, PSN and Zener related texture modifications were observed.
- When comparing different Zener pinning models with the experimental data, it has been shown that in the original Smith–Zener approach the dispersoid volume fraction is to be weighted with the exponent 0.33 (third root).
- Coarse primary phase particles can affect the microstructure by both PSN and pinning, although the effects are only well observable in combination with low fractions of dispersoids.

## Figures and Tables

**Table 1:** Analyzed texture components with respective ideal orientations and Euler angles [76,77].

Component	{hkl}<uvw>	$\varphi_{1},\Phi ,\varphi_{2}$
Brass	{011}<211>	35.3, 45, 0
		90, 144.7, 225
Copper	{112}<111>	180, 21.8, 0
		289.5, 45, 0
S	{123}<634>	121, 36.7, 26.6
		302.3, 18.4, 0
		301, 36.7, 26.6
		122.3, 18.4, 0
Cube	{001}<100>	0, 0, 0
Goss	{011}<100>	0, 45, 0
P	{011}<122>	90, 35.3, 45
		0, 21.8, 360
Cube_ND_	{001}<310>	22, 0, 0

**Table 2:** Grain size results for 63% CRD samples from LOM, BSE and EBSD images.

Alloy	Cast_Homogenization	R_LOM_ [µm]		R_BSE_ [µm]		R_EBSD_ [µm]	
		RD	ND	RD	ND	RD	ND
LFe-LMn	NR-C_500 °C	17.4 ± 3.8	16.0 ± 3.1	16.2 ± 0.6	13.8 ± 1.0	14.3 ± 8.1	12.5 ± 6.7
	S-C_500 °C	22.8 ± 4.9	18.7 ± 2.3	24.7 ± 1.1	21.1 ± 1.3	18.8 ± 12.5	19.7 ± 12.4
	NR-C_550 °C	19.6 ± 1.4	13.7 ± 1.1	14.2 ± 0.3	14.1 ± 0.5	13.5 ± 7.9	11.6 ± 6.3
	S-C_550 °C	18.8 ± 1.5	16.4 ± 3.0	18.4 ± 0.8	17.7 ± 1.2	16.3 ± 9.9	15.8 ± 9.3
LFe-HMn	NR-C_500 °C	6.5 ± 0.7	5.5 ± 0.5	6.7 ± 0.2	5.3 ± 0.3	6.0 ± 2.8	5.5 ± 2.3
	S-C_500 °C	12.1 ± 1.3	7.5 ± 0.5	10.2 ± 0.6	7.3 ± 0.5	11.5 ± 8.3	7.1 ± 3.8
	NR-C_550 °C	7.0 ± 0.8	5.4 ± 0.4	6.1 ± 0.3	5.3 ± 0.4	5.9 ± 2.6	5.9 ± 2.6
	S-C_550 °C	8.4 ± 0.9	7.2 ± 0.4	8.6 ± 0.4	7.1 ± 0.4	7.0 ± 3.7	5.8 ± 2.7
HFe-LMn	NR-C_500 °C	10.0 ± 1.1	10.8 ± 1.4	13.4 ± 1.1	8.8 ± 1.0	8.3 ± 4.9	8.0 ± 4.4
	S-C_500 °C	17.0 ± 0.8	15.9 ± 1.6	17.8 ± 0.8	17.9 ± 0.8	15.1 ± 9.4	14.1 ± 8.0
	NR-C_550 °C	10.4 ± 0.9	9.5 ± 1.1	8.4 ± 0.8	9.3 ± 0.6	9.1 ± 5.1	9.3 ± 5.1
	S-C_550 °C	14.7 ± 2.7	15.0 ± 2.6	13.8 ± 0.8	15.0 ± 0.8	11.5 ± 7.1	12.8 ± 8.6
HFe-HMn	NR-C_500 °C	8.8 ± 1.4	5.6 ± 0.7	6.7 ± 0.9	5.3 ± 0.2	6.3 ± 3.1	5.0 ± 2.0
	S-C_500 °C	10.7 ± 1.4	7.1 ± 0.7	9.1 ± 0.2	6.4 ± 0.3	7.3 ± 4.1	5.9 ± 2.7
	NR-C_550 °C	6.6 ± 0.6	5.3 ± 0.6	5.6 ± 0.6	5.4 ± 0.5	5.5 ± 2.3	4.6 ± 1.6
	S-C_550 °C	9.7 ± 2.0	7.6 ± 1.5	9.0 ± 0.4	7.2 ± 0.5	7.2 ± 3.9	6.0 ± 2.9

Cast_Homogenization: casting conditions and applied homogenization temperature; R_LOM_, R_BSE_, R_EBSD_: calculated grain radii from results of the line intercept measurements and correction factors; RD, ND: grain size in rolling and normal direction.

**Table 3:** Grain size results for 35% CRD samples from LOM, BSE and EBSD images.

Alloy	Cast_Homogenization	R_LOM_ [µm]		R_BSE_ [µm]		R_EBSD_ [µm]	
		RD	ND	RD	ND	RD	ND
LFe-LMn	NR-C_500 °C	18.6 ± 0.4	18.9 ± 3.2	19.5 ± 0.6	20.0 ± 0.6	17.2 ± 10.3	17.1 ± 9.9
	S-C_500 °C	21.5 ± 3.7	19.3 ± 2.3	25.1 ± 2.2	22.4 ± 2.0	20.7 ± 13.2	20.2 ± 12.5
	NR-C_550 °C	18.8 ± 1.5	18.2 ± 3.8	16.3 ± 0.9	16.4 ± 0.7	14.7 ± 8.6	14.0 ± 7.7
	S-C_550 °C	22.9 ± 3.2	19.5 ± 3.4	22.7 ± 1.7	21.9 ± 1.2	18.3 ± 10.6	16.8 ± 9.5
LFe-HMn	NR-C_500 °C	12.6 ± 2.3	10.9 ± 2.4	9.3 ± 0.5	7.0 ± 0.7	8.9 ± 5.5	6.4 ± 3.2
	S-C_500 °C	15.0 ± 2.6	10.2 ± 1.1	19.4 ± 2.5	14.2 ± 0.3	11.5 ± 8.3	7.1 ± 3.8
	NR-C _550 °C	12.5 ± 2.2	8.7 ± 0.6	10.0 ± 1.0	7.3 ± 0.7	8.9 ± 5.4	6.6 ± 3.4
	S-C_550 °C	13.7 ± 2.3	10.1 ± 0.7	12.4 ± 0.4	10.1 ± 0.4	10.2 ± 6.8	7.9 ± 4.5
HFe-LMn	NR-C_500 °C	12.1 ± 1.2	12.0 ± 1.9	13.2 ± 0.6	12.6 ± 1.0	10.4 ± 5.8	9.2 ± 4.8
	S-C_500 °C	18.4 ± 3.0	16.7 ± 2.5	20.9 ± 1.5	19.1 ± 1.4	17.3 ± 10.5	16.4 ± 9.9
	NR-C_550 °C	13.0 ± 2.2	13.6 ± 1.2	13.0 ± 0.8	13.9 ± 0.7	11.1 ± 6.3	9.9 ± 5.2
	S-C_550 °C	15.2 ± 3.2	15.6 ± 2.9	17.4 ± 0.6	17.4 ± 0.4	13.4 ± 7.8	12.1 ± 6.8
HFe-HMn	NR-C_500 °C	10.7 ± 1.6	7.2 ± 0.8	7.8 ± 1.1	6.0 ± 0.4	7.0 ± 3.8	5.4 ± 2.3
	S-C_500 °C	17.7 ± 3.8	9.6 ± 0.8	11.6 ± 1.8	9.0 ± 0.8	10.2 ± 7.2	7.4 ± 4.0
	NR-C_550 °C	9.1 ± 1.4	7.3 ± 0.4	9.0 ± 0.4	7.9 ± 0.6	7.2 ± 3.8	6.0 ± 2.8
	S-C_550 °C	14.9 ± 2.3	10.6 ± 1.4	11.1 ± 0.9	8.8 ± 1.0	9.2 ± 5.8	7.7 ± 4.2

Cast_Homogenization: casting conditions and applied homogenization temperature; R_LOM_, R_BSE_, R_EBSD_: calculated grain radii from results of the line intercept measurements and correction factors; RD, ND: grain size in rolling and normal direction.

**Table 4:** Volume fractions and morphological parameters of the dispersoids for NR-C cast samples homogenized at 500 °C, cold rolled to a CRD of 63% and soft annealed; limiting grain sizes calculated using the models of Smith–Zener [26], Manohar [33] and Ryum [32].

Sample	Volume Fraction (vol. %)	r (nm)	AR	R_lim_ (µm)			
				Smith–Zener	Manohar	Ryum RD	Ryum ND
LFe-LMn	0.01	50	1.54	1207.0	153.9	1775.5	1094.5
LFe-HMn	1.01	73	2.13	9.6	1.2	19.42	7.95
HFe-LMn	0.08	67	1.95	105.8	13.5	195.3	89.7
HFe-HMn	1.07	63	2.06	7.9	1.0	15.4	6.6

r: average dispersoid radius; AR: aspect ratio of the ellipsoidal particles; R_lim_: calculated limiting grain radius using different Zener pinning model parameters.

**Table 5:** Volume fractions and morphological parameters of the dispersoids for NR-C cast samples homogenized at 550 °C, cold rolled to a CRD of 63% and soft annealed; limiting grain sizes calculated using the models of Smith–Zener [26], Manohar [33] and Ryum [32].

Sample	Volume Fraction (vol. %)	r (nm)	AR	R_lim_ (µm)			
				Smith–Zener	Manohar	Ryum RD	Ryum ND
LFe-LMn	0.04	65	1.60	199.6	25.5	304.2	179.2
LFe-HMn	0.58	81	2.15	18.6	2.4	37.8	15.3
HFe-LMn	0.06	76	1.65	169.0	21.5	264.4	150.5
HFe-HMn	1.00	79	1.93	10.5	1.34	19.2	8.9

r: average dispersoid radius; AR: aspect ratio of the ellipsoidal particles; R_lim_: calculated limiting grain radius using different Zener pinning model parameters.

**Figure A1:**
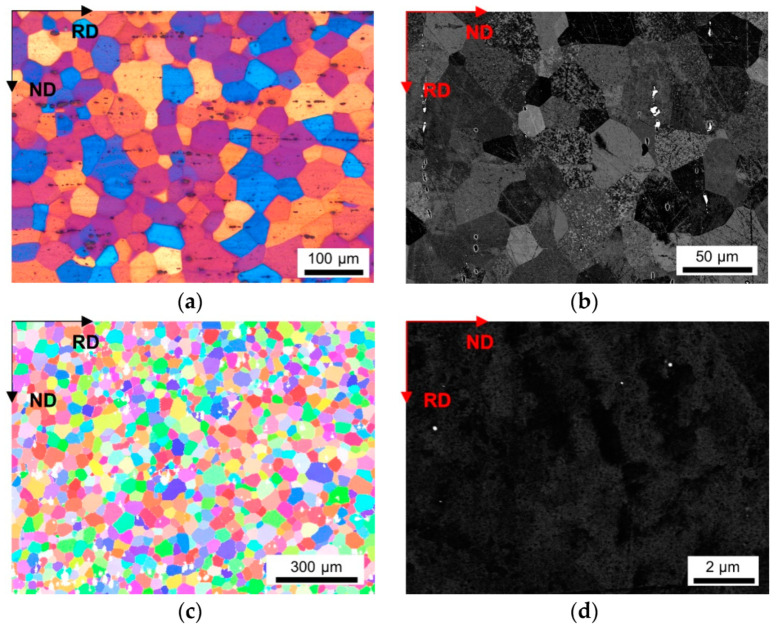
Microstructure of the soft annealed LFe-LMn cast under S-C condition, homogenized at 500 °C and cold rolled to a CRD of 35%. (**a**) LOM. (**b**) BSE image. (**c**) EBSD IPF map in RD–ND plane. (**d**) BSE micrograph showing dispersoids.

**Figure A2:**
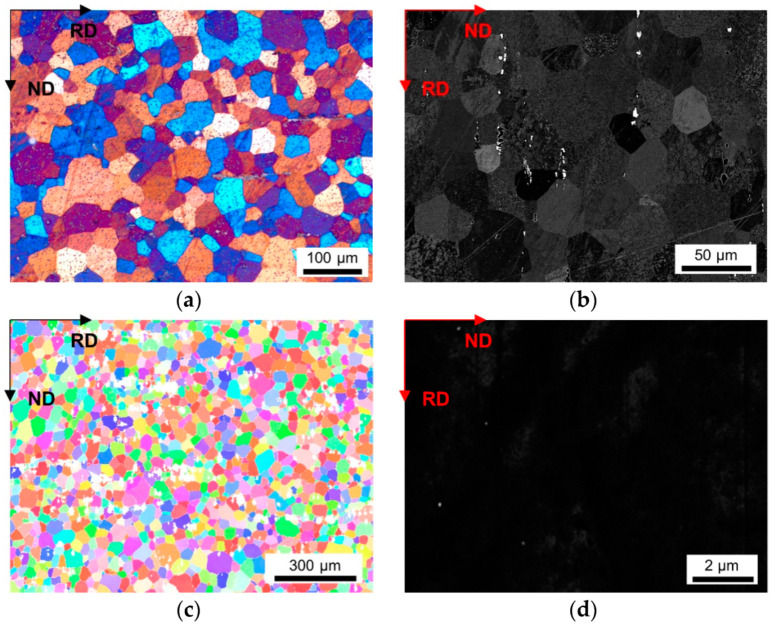
Microstructure of the soft annealed LFe-LMn cast under S-C condition, homogenized at 500 °C and cold rolled to a CRD of 63%. (**a**) LOM. (**b**) BSE image. (**c**) EBSD IPF map in RD–ND plane. (**d**) BSE micrograph showing dispersoids.

**Figure A3:**
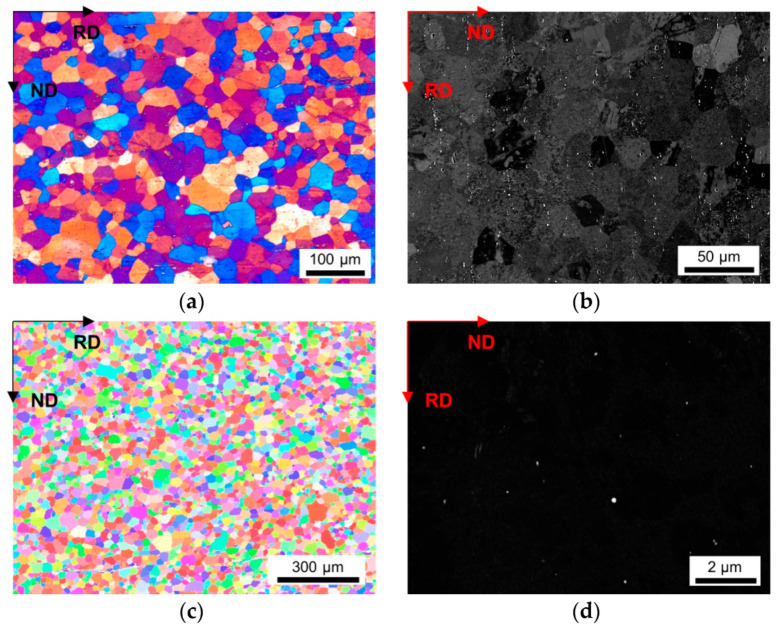
Microstructure of the soft annealed LFe-LMn cast under NR-C condition, homogenized at 500 °C and cold rolled to a CRD of 63%. (**a**) LOM. (**b**) BSE image. (**c**) EBSD IPF map in RD–ND plane. (**d**) BSE micrograph showing dispersoids.

**Figure A4:**
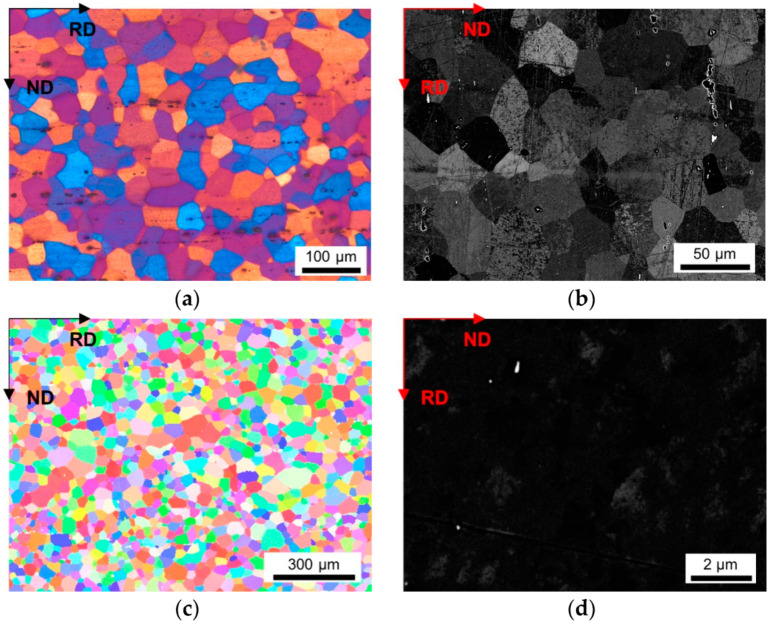
Microstructure of the soft annealed LFe-LMn cast under S-C condition, homogenized at 550 °C and cold rolled to a CRD of 35%. (**a**) LOM. (**b**) BSE image. (**c**) EBSD IPF map in RD–ND plane. (**d**) BSE micrograph showing dispersoids.

**Figure A5:**
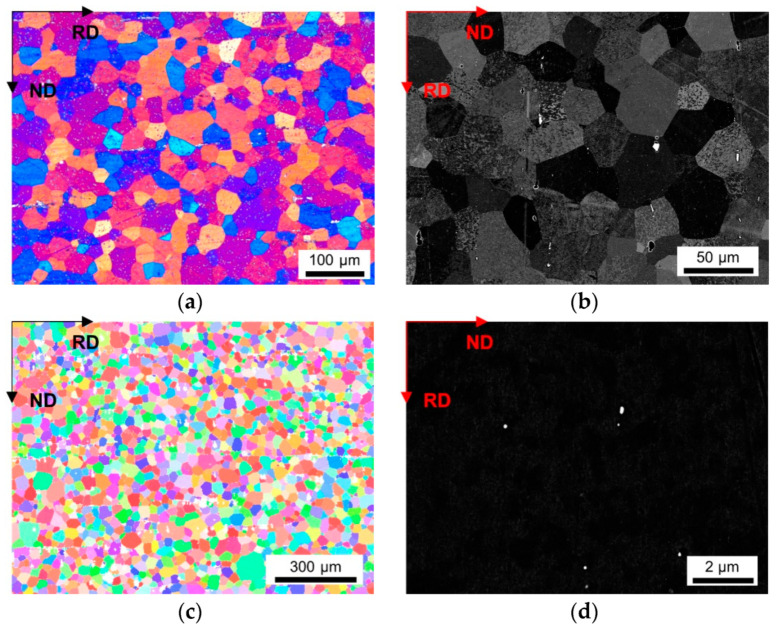
Microstructure of the soft annealed LFe-LMn cast under S-C condition, homogenized at 550 °C and cold rolled to a CRD of 63%. (**a**) LOM. (**b**) BSE image. (**c**) EBSD IPF map in RD–ND plane. (**d**) BSE micrograph showing dispersoids.

**Figure A7:**
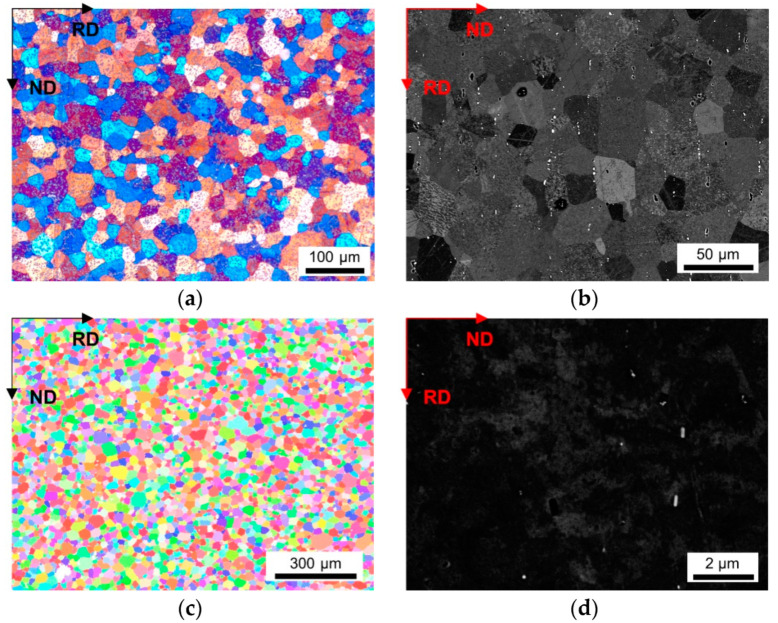
Microstructure of the soft annealed LFe-LMn cast under NR-C condition, homogenized at 550 °C and cold rolled to a CRD of 63%. (**a**) LOM. (**b**) BSE image. (**c**) EBSD IPF map in RD–ND plane. (**d**) BSE micrograph showing dispersoids.

**Figure A8:**
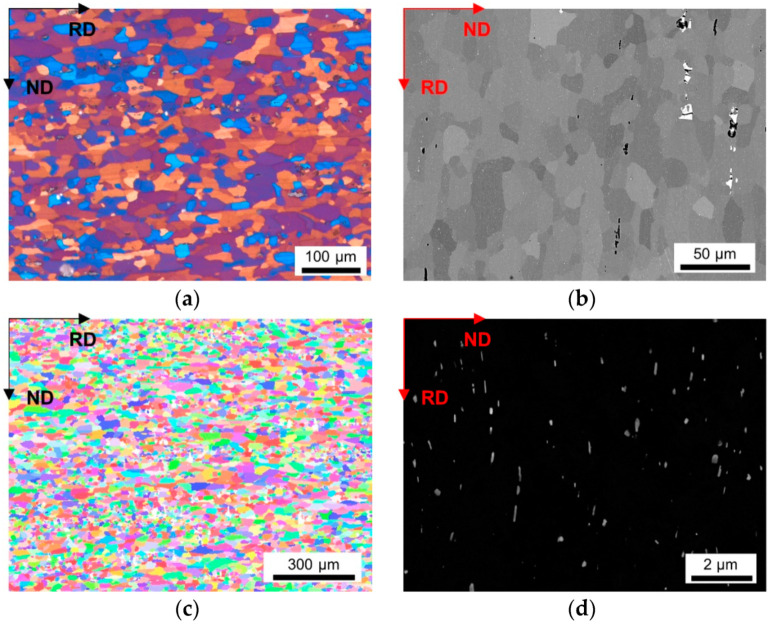
Microstructure of the soft annealed LFe-HMn cast under S-C condition, homogenized at 500 °C and cold rolled to a CRD of 35%. (**a**) LOM. (**b**) BSE image. (**c**) EBSD IPF map in RD–ND plane. (**d**) BSE micrograph showing dispersoids.

**Figure A9:**
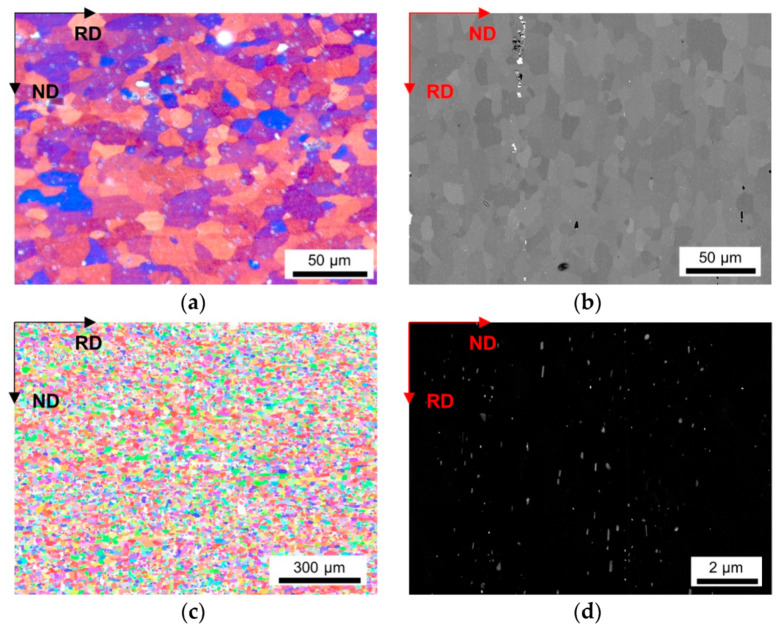
Microstructure of the soft annealed LFe-HMn cast under S-C condition, homogenized at 500 °C and cold rolled to a CRD of 63%. (**a**) LOM. (**b**) BSE image. (**c**) EBSD IPF map in RD–ND plane. (**d**) BSE micrograph showing dispersoids.

**Figure A10:**
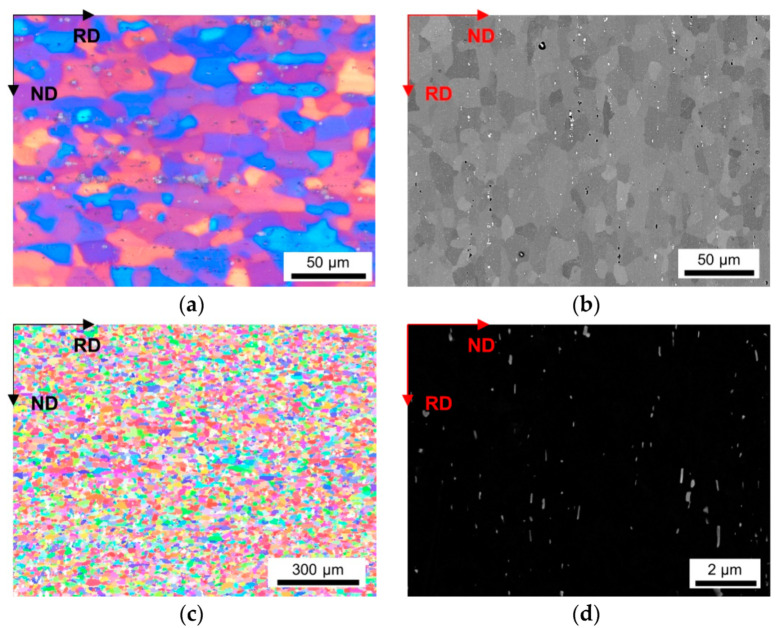
Microstructure of the soft annealed LFe-HMn cast under NR-C condition, homogenized at 500 °C and cold rolled to a CRD of 35%. (**a**) LOM. (**b**) BSE image. (**c**) EBSD IPF map in RD–ND plane. (**d**) BSE micrograph showing dispersoids.

**Figure A11:**
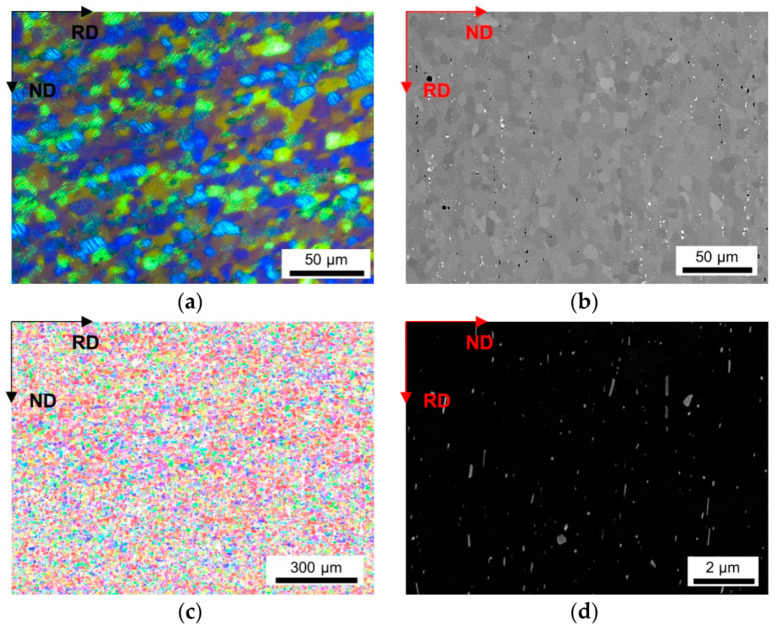
Microstructure of the soft annealed LFe-HMn cast under NR-C conditions, homogenized at 500 °C and cold rolled to a CRD of 63%. (**a**) LOM. (**b**) BSE image. (**c**) EBSD IPF map in RD–ND plane. (**d**) BSE micrograph showing dispersoids.

**Figure A12:**
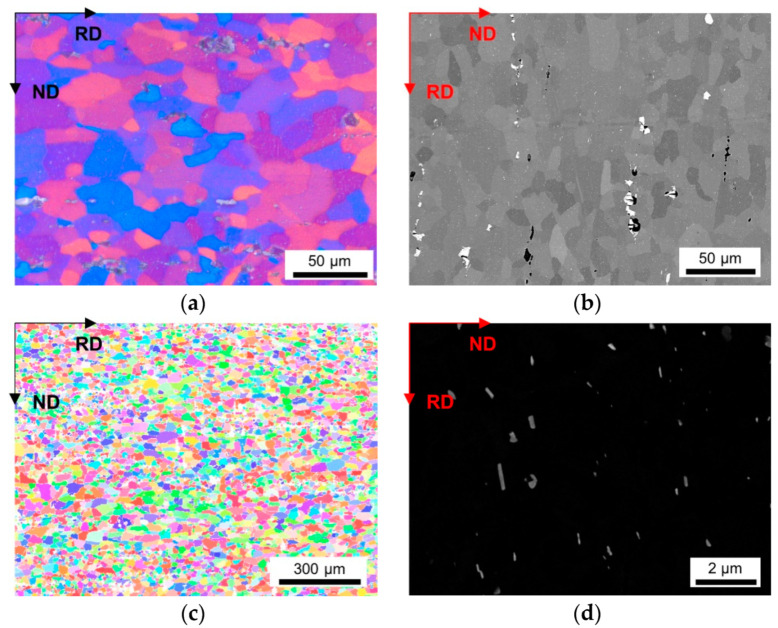
Microstructure of the soft annealed LFe-HMn cast under S-C conditions, homogenized at 550 °C and cold rolled to a CRD of 35%. (**a**) LOM. (**b**) BSE image. (**c**) EBSD IPF map in RD–ND plane. (**d**) BSE micrograph showing dispersoids.

**Figure A13:**
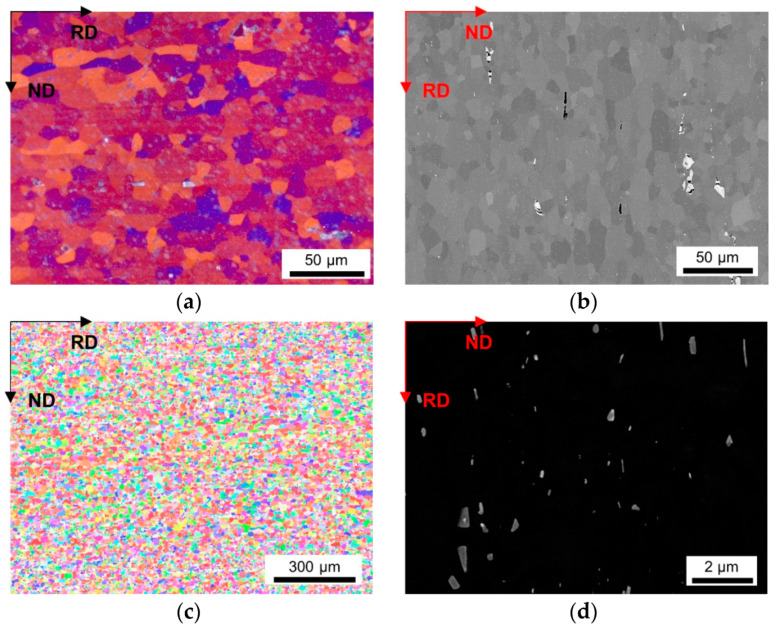
Microstructure of the soft annealed LFe-HMn cast under S-C conditions, homogenized at 550 °C and cold rolled to a CRD of 63%. (**a**) LOM. (**b**) BSE image. (**c**) EBSD IPF map in RD–ND plane. (**d**) BSE micrograph showing dispersoids.

**Figure A14:**
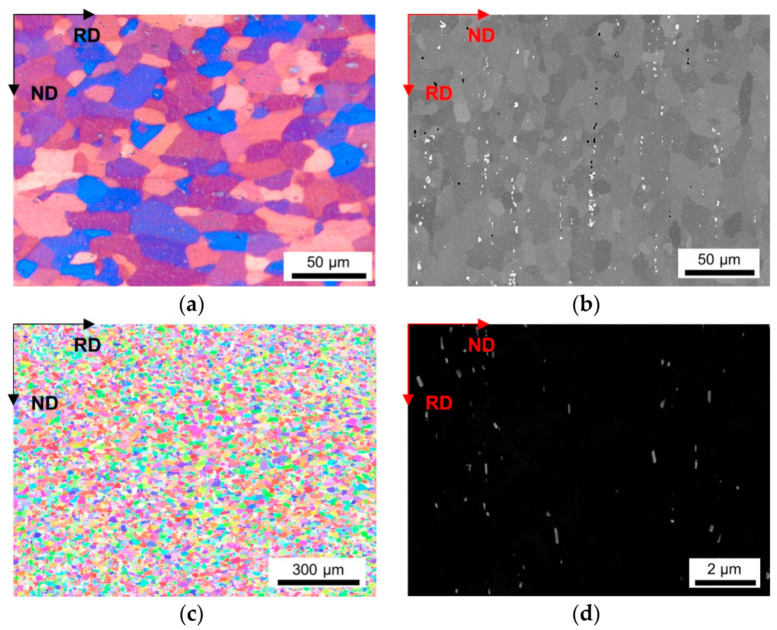
Microstructure of the soft annealed LFe-HMn cast under NR-C conditions, homogenized at 550 °C and cold rolled to a CRD of 35%. (**a**) LOM. (**b**) BSE image. (**c**) EBSD IPF map in RD–ND plane. (**d**) BSE micrograph showing dispersoids.

**Figure A15:**
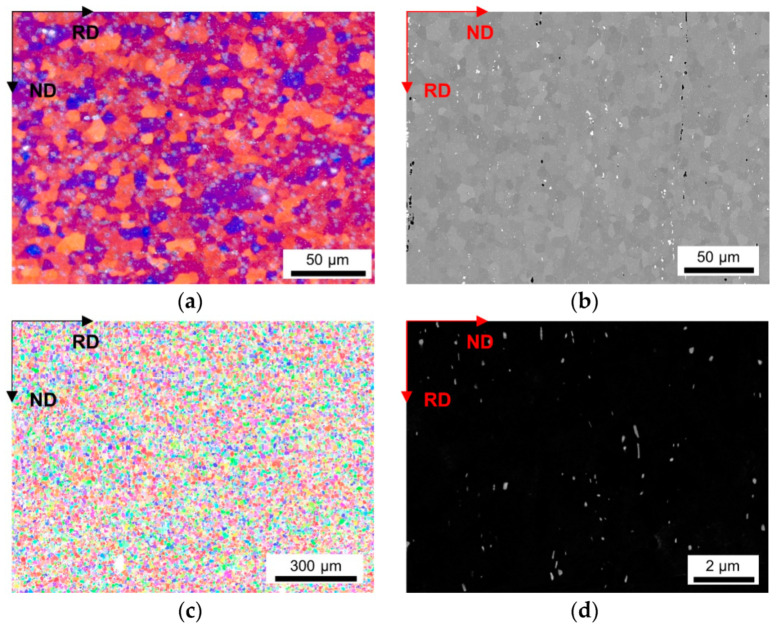
Microstructure of the soft annealed LFe-HMn cast under NR-C conditions, homogenized at 550 °C and cold rolled to a CRD of 63%. (**a**) LOM. (**b**) BSE image. (**c**) EBSD IPF map in RD–ND plane. (**d**) BSE micrograph showing dispersoids.

**Figure A16:**
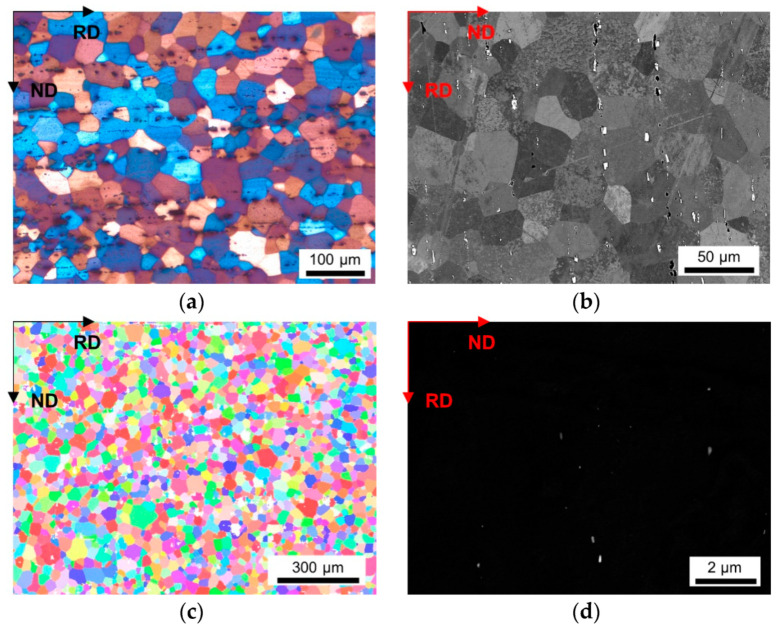
Microstructure of the soft annealed HFe-LMn cast under S-C conditions, homogenized at 500 °C and cold rolled to a CRD of 35%. (**a**) LOM. (**b**) BSE image. (**c**) EBSD IPF map in RD–ND plane. (**d**) BSE micrograph showing dispersoids.

**Figure A17:**
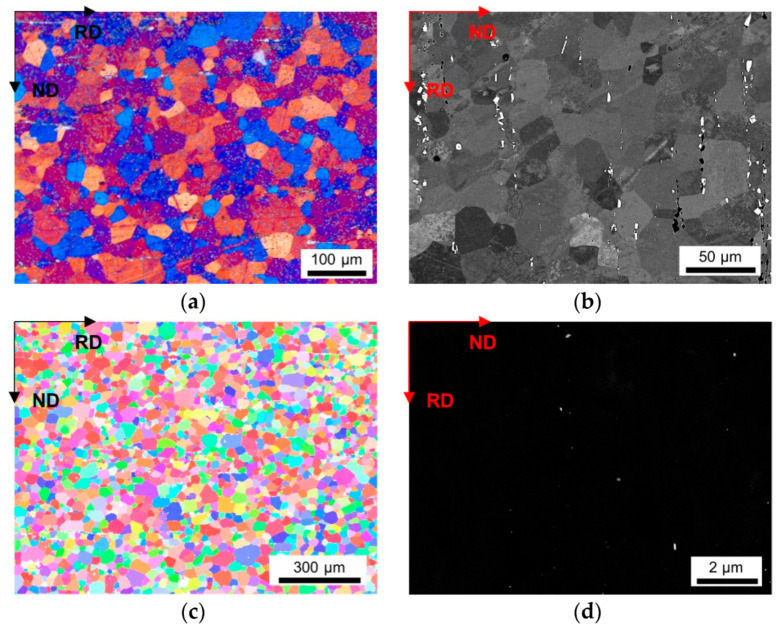
Microstructure of the soft annealed HFe-LMn cast under S-C conditions, homogenized at 500 °C and cold rolled to a CRD of 63%. (**a**) LOM. (**b**) BSE image. (**c**) EBSD IPF map in RD–ND plane. (**d**) BSE micrograph showing dispersoids.

**Figure A18:**
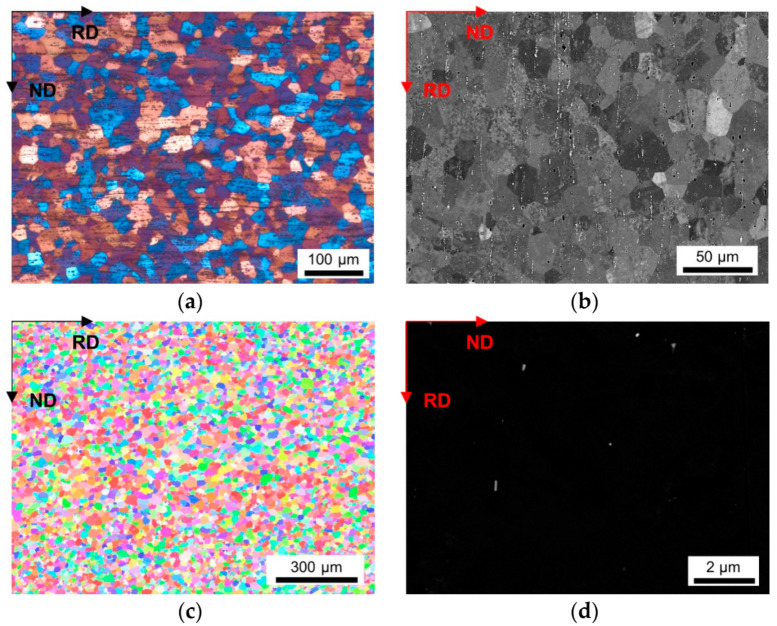
Microstructure of the soft annealed HFe-LMn cast under NR-C conditions, homogenized at 500 °C and cold rolled to a CRD of 35%. (**a**) LOM. (**b**) BSE image. (**c**) EBSD IPF map in RD–ND plane. (**d**) BSE micrograph showing dispersoids.

**Figure A19:**
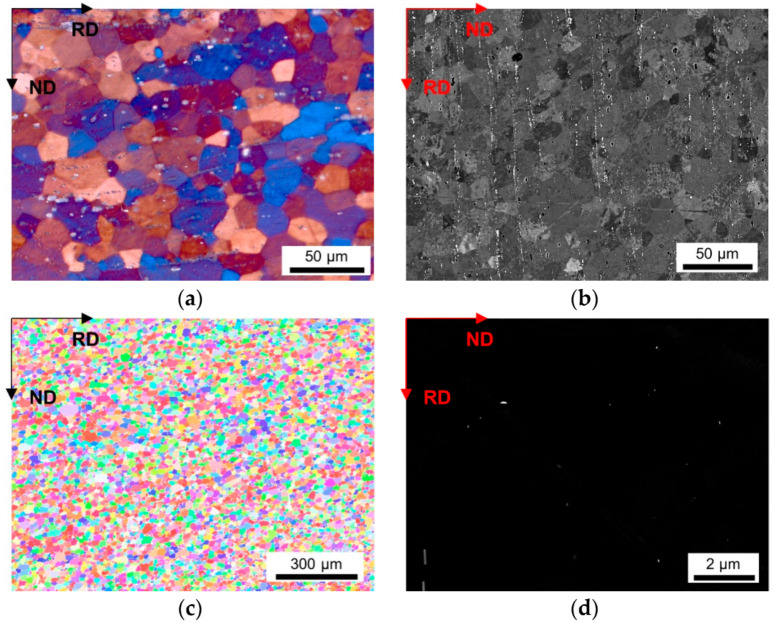
Microstructure of the soft annealed HFe-LMn cast under NR-C conditions, homogenized at 500 °C and cold rolled to a CRD of 63%. (**a**) LOM. (**b**) BSE image. (**c**) EBSD IPF map in RD–ND plane. (**d**) BSE micrograph showing dispersoids.

**Figure A20:**
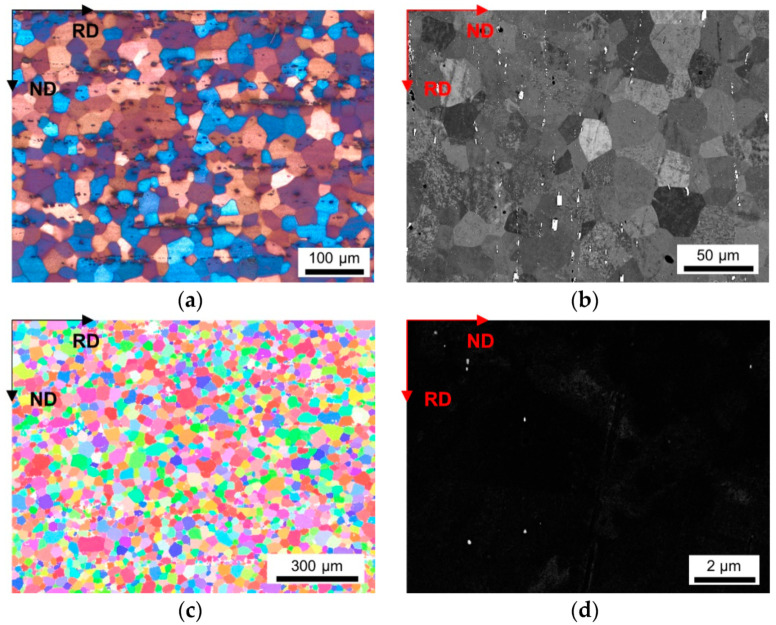
Microstructure of the soft annealed HFe-LMn cast under S-C conditions, homogenized at 550 °C and cold rolled to a CRD of 35%. (**a**) LOM. (**b**) BSE image. (**c**) EBSD IPF map in RD–ND plane. (**d**) BSE micrograph showing dispersoids.

**Figure A21:**
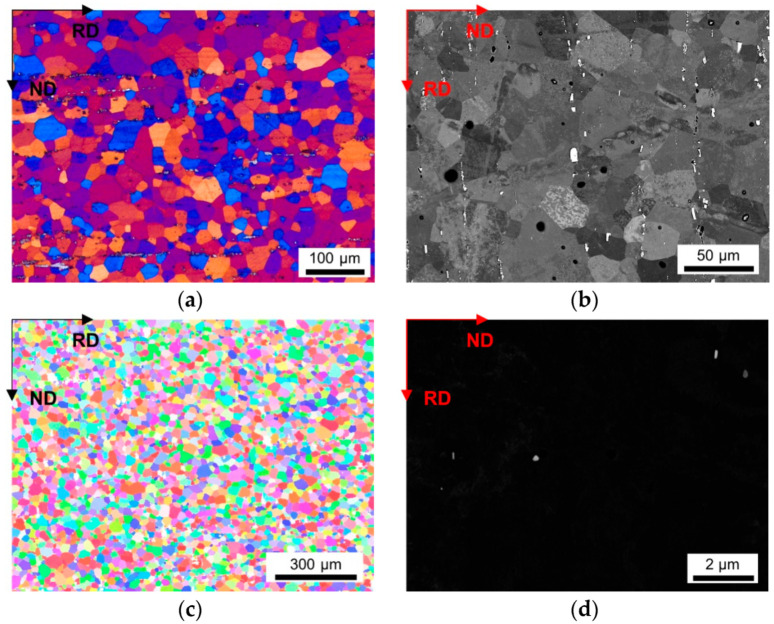
Microstructure of the soft annealed HFe-LMn cast under S-C conditions, homogenized at 550 °C and cold rolled to a CRD of 63%. (**a**) LOM. (**b**) BSE image. (**c**) EBSD IPF map in RD–ND plane. (**d**) BSE micrograph showing dispersoids.

**Figure A22:**
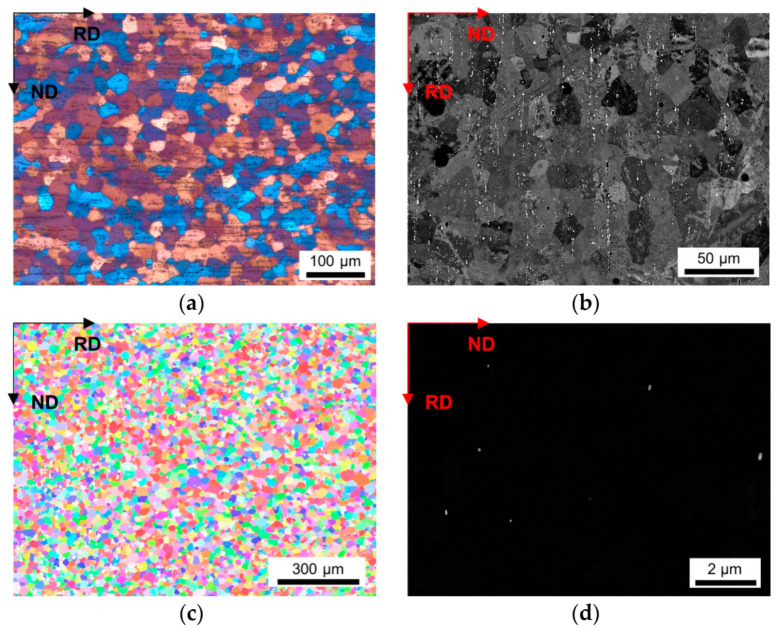
Microstructure of the soft annealed HFe-LMn cast under NR-C conditions, homogenized at 550 °C and cold rolled to a CRD of 35%. (**a**) LOM. (**b**) BSE image. (**c**) EBSD IPF map in RD–ND plane. (**d**) BSE micrograph showing dispersoids.

**Figure A23:**
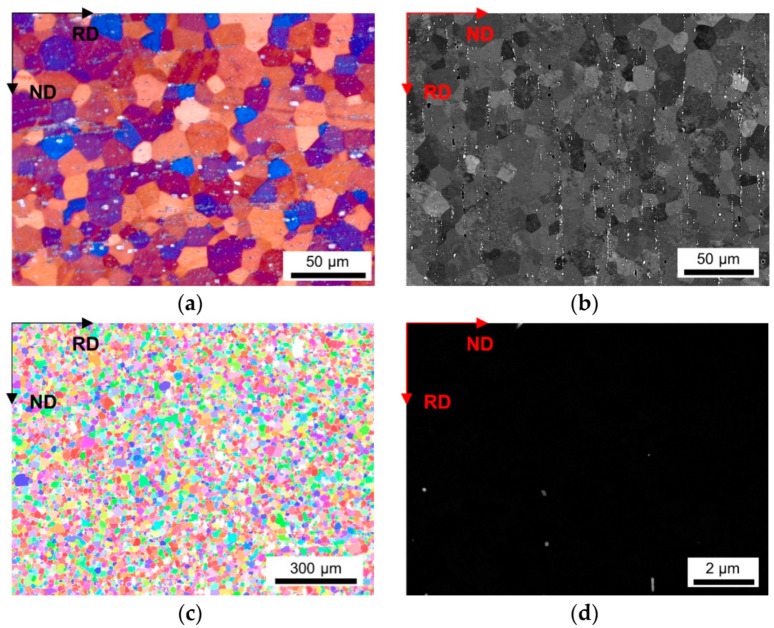
Microstructure of the soft annealed HFe-LMn cast under NR-C conditions, homogenized at 550 °C and cold rolled to a CRD of 63%. (**a**) LOM. (**b**) BSE image. (**c**) EBSD IPF map in RD–ND plane. (**d**) BSE micrograph showing dispersoids.

**Figure A24:**
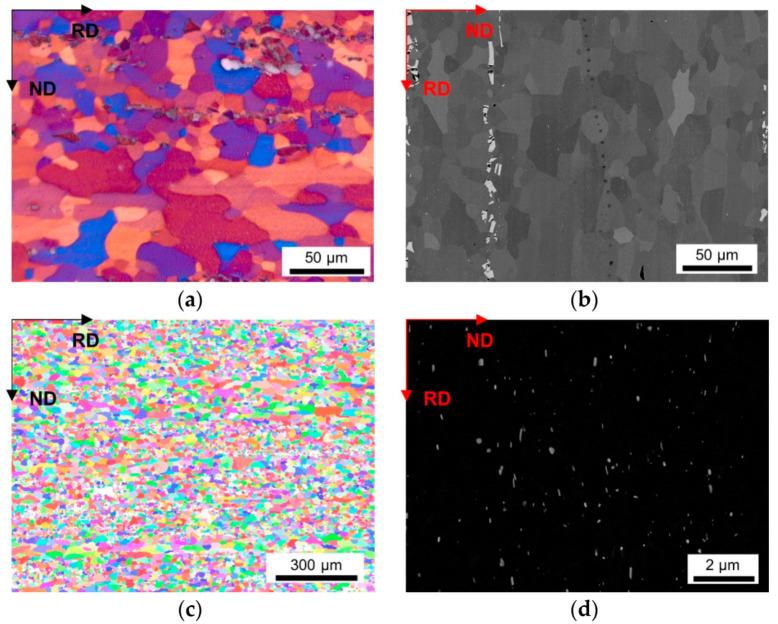
Microstructure of the soft annealed HFe-HMn cast under S-C conditions, homogenized at 500 °C and cold rolled to a CRD of 35%. (**a**) LOM. (**b**) BSE image. (**c**) EBSD IPF map in RD–ND plane. (**d**) BSE micrograph showing dispersoids.

**Figure A25:**
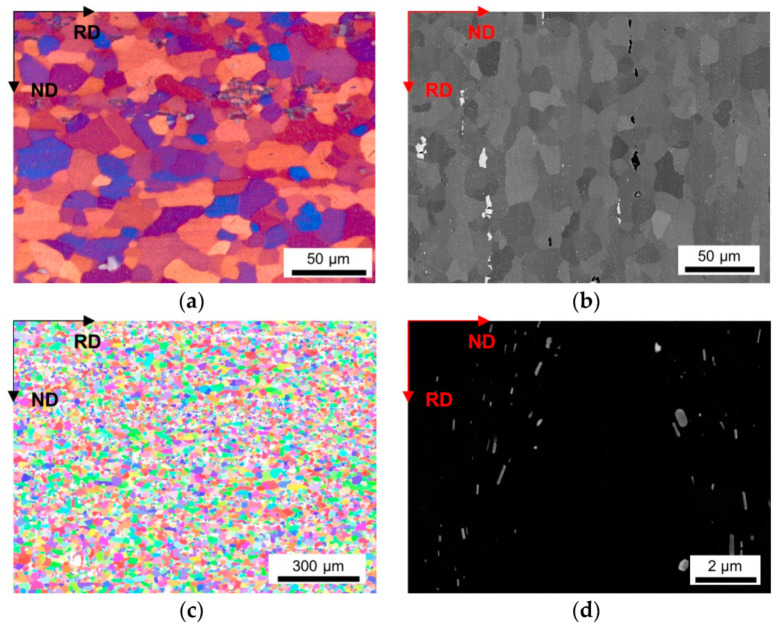
Microstructure of the soft annealed HFe-HMn cast under S-C conditions, homogenized at 550 °C and cold rolled to a CRD of 35%. (**a**) LOM. (**b**) BSE image. (**c**) EBSD IPF map in RD–ND plane. (**d**) BSE micrograph showing dispersoids.

**Figure A26:**
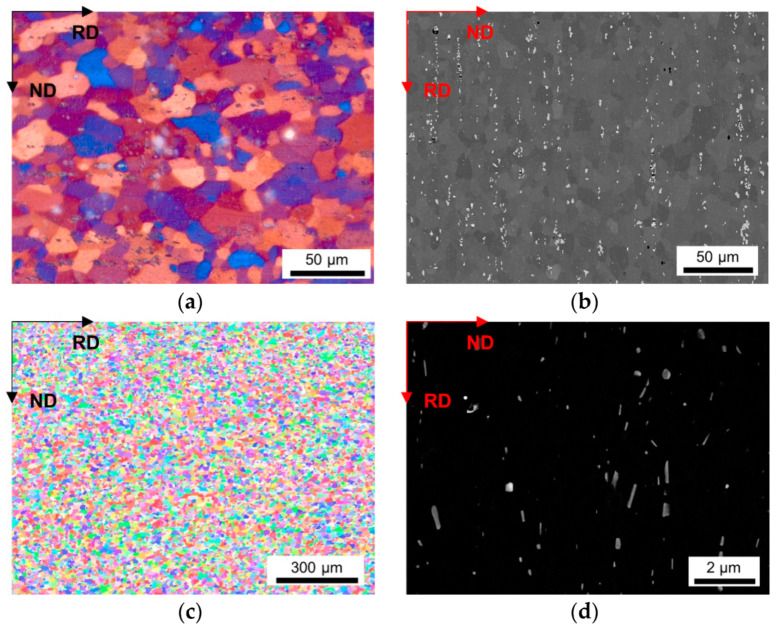
Microstructure of the soft annealed HFe-HMn cast under NR-C conditions, homogenized at 550 °C and cold rolled to a CRD of 35%. (**a**) LOM. (**b**) BSE image. (**c**) EBSD IPF map in RD–ND plane. (**d**) BSE micrograph showing dispersoids.

**Figure A27:**
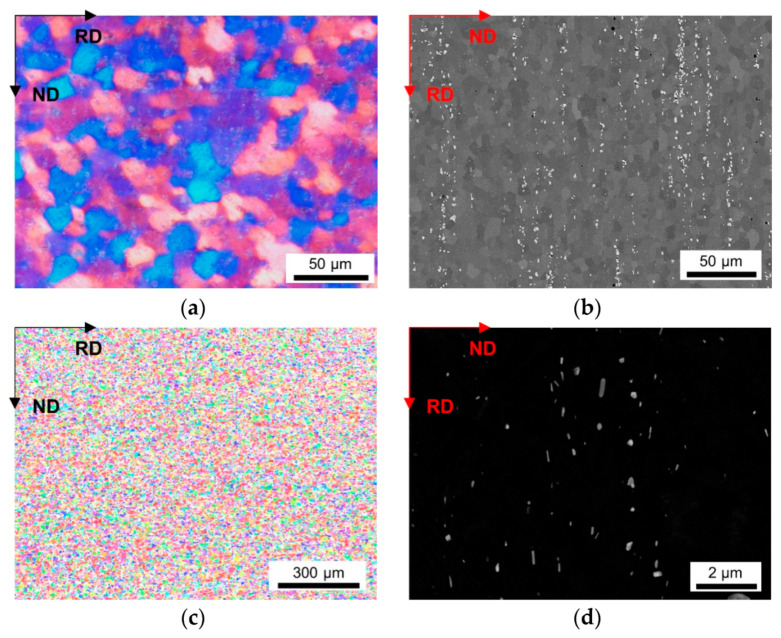
Microstructure of the soft annealed HFe-HMn cast under NR-C conditions, homogenized at 550 °C and cold rolled to a CRD of 63%. (**a**) LOM. (**b**) BSE image. (**c**) EBSD IPF map in RD–ND plane. (**d**) BSE micrograph showing dispersoids.

## Appendix A

**Table A1:** Volume fractions and morphological parameters of the dispersoids for S-C cast samples homogenized at 500 °C, cold rolled to a CRD of 35% and soft annealed; limiting grain sizes calculated using the models of Smith–Zener [26], Manohar [33] and Ryum [32].

Sample	Volume Fraction [vol. %]	r [nm]	AR	R_lim_ [µm]			
				Smith–Zener	Manohar	Ryum RD	Ryum ND
LFe-LMn	0.01	78	1.67	1105.8	141.0	1753.0	981.3
LFe-HMn	0.96	81	2.02	11.3	1.4	21.5	9.5
HFe-LMn	0.01	45	1.77	462.8	59.0	773.9	404.2
HFe-HMn	0.64	62	1.82	12.8	1.63	22.1	11.1

r: average dispersoid radius; AR: aspect ratio of the ellipsoidal particles; R_lim_: calculated limiting grain radius using different Zener pinning model parameters.

**Table A2:** Volume fractions and morphological parameters of the dispersoids for S-C cast samples homogenized at 550 °C, cold rolled to a CRD of 35% and soft annealed; limiting grain sizes calculated using the models of Smith–Zener [26], Manohar [33] and Ryum [32].

Sample	Volume Fraction [vol. %]	r [nm]	AR	R_lim_ [µm]			
				Smith–Zener	Manohar	Ryum RD	Ryum ND
LFe-LMn	0.03	75	1.81	293.5	37.4	503.3	254.4
LFe-HMn	0.64	82	1.88	17.13	2.2	30.4	14.7
HFe-LMn	0.01	72	1.70	734.4	93.6	1183.9	648.5
HFe-HMn	1.35	78	1.91	7.7	1.0	13.9	6.6

r: average dispersoid radius; AR: aspect ratio of the ellipsoidal particles; R_lim_: calculated limiting grain radius using different Zener pinning model parameters.
